# Design and Evolution of Enhanced Peptide–Peptide Ligation for Modular Transglutaminase Assembly

**DOI:** 10.1021/acs.bioconjchem.3c00122

**Published:** 2023-06-08

**Authors:** Anthony H. Keeble, Dominic P. Wood, Mark Howarth

**Affiliations:** † Department of Biochemistry, University of Oxford, South Parks Road, Oxford OX1 3QU, U.K.; ‡ Department of Pharmacology, University of Cambridge, Tennis Court Road, Cambridge CB2 1PD, U.K.

## Abstract

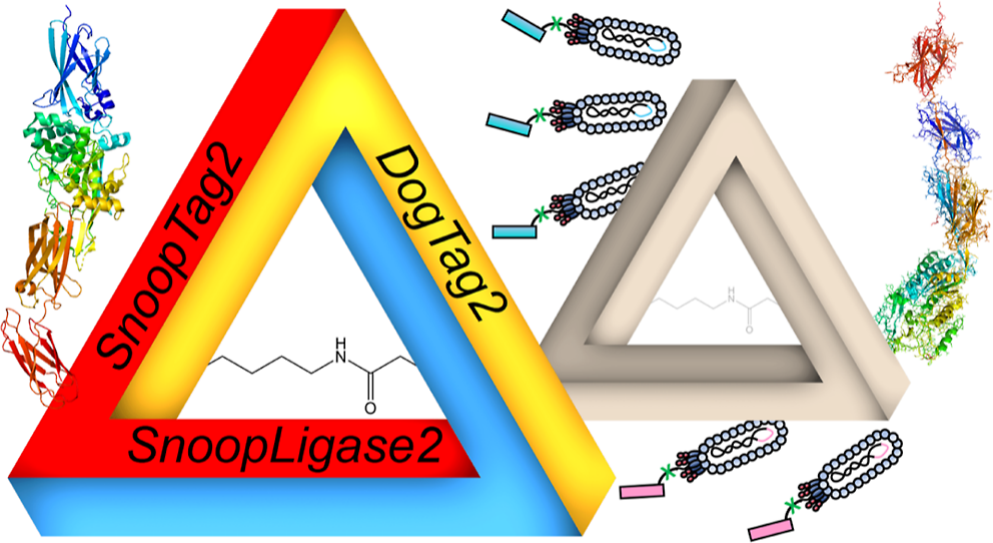

Robust and precise tools are needed to enhance the functionality and resilience of synthetic nanoarchitectures. Here, we have employed directed evolution and rational design to build a fast-acting molecular superglue from a bacterial adhesion protein. We have generated the SnoopLigase2 coupling system, a genetically encoded route for efficient transamidation between SnoopTag2 and DogTag2 peptides. Each peptide was selected for rapid reaction by phage display screening. The optimized set allows more than 99% completion and is compatible with diverse buffers, pH values, and temperatures, accelerating the reaction over 1000-fold. SnoopLigase2 directs a specific reaction in the mammalian secretory pathway, allowing covalent display on the plasma membrane. Transglutaminase 2 (TG2) has a network of interactions and substrates amidst the mammalian cell surface and extracellular matrix. We expressed a modified TG2 with resistance to oxidative inactivation and minimal self-reactivity. SnoopLigase2 enables TG2 functionalization with transforming growth factor alpha (TGFα) in routes that would be impossible through genetic fusion. The TG2:TGFα conjugate retained transamidase activity, stably anchored TGFα for signal activation in the extracellular environment, and reprogrammed cell behavior. This modular toolbox should create new opportunities for molecular assembly, both for novel biomaterials and complex cellular environments.

## Introduction

**Figure 1:**
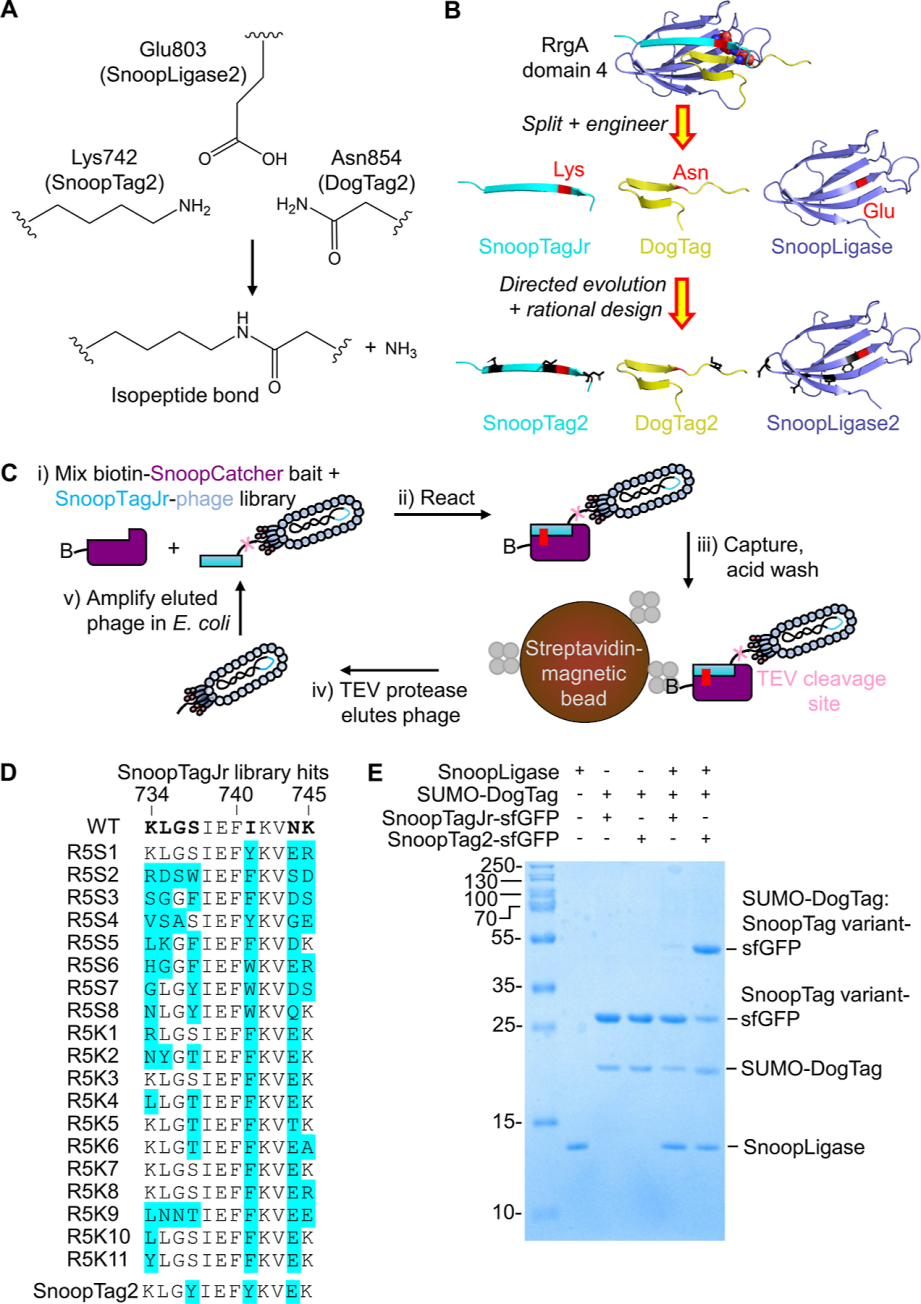
Directed evolution of SnoopTag2. (A) SnoopLigase2 promotes the transamidation of Lys on SnoopTag2 with Asn on DogTag2. (B) Cartoon of the optimization of domain 4 of RrgA (PDB 2WW8) to produce the initial SnoopLigase set and then SnoopTag2 (cyan), DogTag2 (yellow), and SnoopLigase2 (dark blue) with mutations in black. Key residues for reaction are marked in red, with the isopeptide bond in spacefill. (C) Schematic of phage display selection for a faster SnoopTagJr variant. A phage library displaying SnoopTagJr variants (not to scale) was panned with biotinylated SnoopCatcher as the bait. B represents biotin, and the small gray circles are streptavidin, with the TEV protease site in pink and the isopeptide bond as a red line. (D) Selected SnoopTagJr hits after five rounds of selection. Residues in bold on the WT sequence indicate the residues mutated, with observed changes in blue. R refers to the selection round; S and K are randomization routes. (E) Enhanced SnoopLigase coupling by SnoopTag2. SnoopTagJr-sfGFP or SnoopTag2-sfGFP was ligated to SUMO-DogTag using SnoopLigase (25 μM each) for 3 h at 21 °C in Tris-borate pH 7.4 (SDS–PAGE with Coomassie staining). A colon indicates that the proteins are ligated together.

Hierarchical structures with stable precise assembly provide extraordinary properties in terms of mechanical strength, optical control, and programming of cellular behavior.^1−3^ New tools for covalent and site-specific ligation are needed, so that we can mimic or surpass such molecular architectures.^4,5^ Various powerful technologies have been developed for peptide–peptide coupling, such as sortase, butelase, connectase, and subtiligase.^6−9^ Linking two components through peptide bonds means that tags are restricted for activity to a particular terminus, but linkage through isopeptide bonds brings the freedom to have tags at either terminus or internal protein sites.^4^ SnoopLigase was previously created to catalyze the coupling of a Lys-containing peptide tag to an Asn-containing peptide tag, generating an isopeptide bond by transamidation (Figure 1A).^10^ SnoopLigase was engineered from a three-way split of domain 4 of the *Streptococcus pneumoniae* adhesin RrgA, followed by protein engineering (Figure 1B). This strategy resulted in SnoopTagJr (containing the reactive Lys), DogTag (containing the reactive Asn), and SnoopLigase (containing the Glu facilitating the reaction) (Figure 1A).^10^ SnoopLigase has found application in enhancing enzyme resilience^10^ and for modular assembly of a malaria vaccine candidate,^11^ viral vectors,^12^ and bispecific nanobodies.^13^ SnoopLigase has the advantage over previous Tag/Catcher ligation systems (SpyTag/SpyCatcher, SnoopTag/SnoopCatcher, and DogTag/DogCatcher) (Figure S1)^14−16^ in that both partners for coupling need only to be fused with a short peptide (SnoopTagJr 12 aa and DogTag 23 aa) rather than a ∼15 kDa protein domain for Catcher-mediated coupling. SnoopLigase^10^ gives higher yield coupling than the isopeptide-forming SpyLigase^17^ and SpyStapler peptide–peptide ligation approaches.^18^ On the other hand, SnoopLigase takes ∼24 h to reach completion with protein concentrations of 10 μM, compared to ∼20 min at protein concentrations of 10 nM for SpyTag003/SpyCatcher003.^10,19^ In part, this decreased rate simply reflects that the probability of three components coming together simultaneously will be lower than for two components. The difference in speed also reflects the role that directed evolution played in accelerating the reactivity of SpyTag003/SpyCatcher003 close to the diffusion limit.^19,20^ The value of directed evolution reflects the unconventional nature of the peptide–protein interaction, where the partners have structural disorder, which makes de novo rational prediction challenging. In addition to its moderate reaction speed, SnoopLigase was limited in the compatible buffers,^10,11^ restricting its wide usage. Here, we employ a combination of directed evolution and rational design to create the SnoopLigase2 system, which reacts more than 1000-fold faster than the original SnoopLigase system and is tolerant of a broad range of reaction conditions.

Microbial transglutaminase from *Streptomyces mobaraensis* has been broadly applied for ligating molecular components, from food production to nanotechnology, taking advantage of its promiscuous reactivity.^21^ Mammalian transglutaminase 2 (TG2, also known as tissue transglutaminase, tTG) is a transamidase with both catalytic and non-catalytic roles in extracellular organization.^22,23^ TG2 has been much less explored for bioconjugation and synthetic biology and may have a narrower range of substrates than microbial transglutaminase, as well as possessing multiple routes for regulation of activity.^22^ TG2 accumulates at the cell surface, contributing to cell adhesion via non-covalent interactions.^24^ TG2 also cross-links extracellular matrix (ECM) proteins via transamidation of Lys and Gln side chains^22^ and is important in a range of diseases, including celiac disease^25^ and cancer metastasis.^23^ TG2 is an attractive candidate for development of a tool to facilitate decoration with recombinant proteins,^26^ both in the extracellular space and for precision modification of surfaces for stable mechanical or optical properties.^1^ To this end, we also engineer here a minimal functional fragment of TG2 for SnoopLigase2 ligation. Transforming growth factor α (TGFα) is a ligand for epidermal growth factor receptor (EGFR).^27^ TGFα-induced activation of EGFR stimulates several downstream signaling pathways, including Erk1/2, Akt, and STAT.^27^ TGFα has a role in cell proliferation^27^ and has been explored as a therapeutic for tissue repair.^28^ We use SnoopLigase2 to facilitate post-translational modular ligation of TGFα to this modified TG2. Robust retention of this TG2:TGFα conjugate at the cell surface was demonstrated, resulting in sustained TGFα activity and programming of cell differentiation.

## Results

### Directed Evolution Selects Faster Reacting SnoopTagJr Variants

Our development of the faster reacting SpyTag/SpyCatcher generations^19,20^ and DogTag/DogCatcher^15^ revealed that directed evolution using phage display is a powerful route for creating faster covalent peptide ligations. Simultaneous improvement of three components in a reaction by selection is difficult. Previous experience on SpyCatcher and DogCatcher systems showed that improvements of the individual components can usually be combined.^15,19,20^ As a first step, we used phage display to select faster reaction of SnoopTagJr variants in the simplest manner by reaction with SnoopCatcher (Figure 1C). SnoopCatcher represents the Catcher from which SnoopTag was originally developed.^14^ M13 phage displaying the SnoopTagJr library as a pIII fusion was incubated with biotinylated SnoopCatcher in solution, followed by capturing the complex on streptavidin-magnetic beads. We disrupted non-covalent complexes by washing at pH 2 and then eluted the reactive phage using Tobacco etch virus (TEV) protease cleavage at a site engineered between the SnoopTagJr variant and pIII (Figure 1C).

SnoopTagJr contains 12 amino acids, which is too long for saturation mutagenesis by phage display. Furthermore, the crystal structure of RrgA domain 4^29^ and previous engineering^10,14,15^ suggested that mutations at SnoopTagJr residues close to the reactive Lys742 or residues packing with the core of the domain were likely to be deleterious. Therefore, we focused our mutations on residues at the N- and C-termini of SnoopTagJr (residues 734–737, 741, and 744–745) (Figure 1D). We also employed different primer libraries with hard randomization (using NNK codons) or soft randomization (using NWW and RVK codons).^30^ We performed rounds of selection with increasing stringency, reducing bait concentration and reaction time, such that in the fifth round, we used only 1 nM bait and 5 min reaction. Clones from the two libraries were picked and sequenced (Figure 1D). The parental residues at positions 734, 735, 736, and 745 were largely conserved. At position 737, aromatic residues (Tyr/Phe/Trp) were selected in the hard randomization library and Ser/Thr in the soft randomization library. We found that aromatic residues were selected at position 741 and negatively charged residues at position 744. After evaluating a range of these leads for SnoopLigase reaction, mutations that were faster reacting or giving equivalent speed but higher polarity were combined to generate SnoopTag2 (Figures 1D and S2). Higher polarity may help the function of peptides as fusion partners.^31^ To compare the coupling efficiency of SnoopTag2 against SnoopTagJr, we fused the tags to superfolder green fluorescent protein (sfGFP). We then used SnoopLigase to direct coupling with DogTag fused to a model domain, small ubiquitin-like modifier (SUMO). SnoopTag2 provided a major improvement in coupling efficiency over SnoopTagJr (Figure 1E).

### Directed Evolution Selects Faster Reacting DogTag Variants

**Figure 2:**
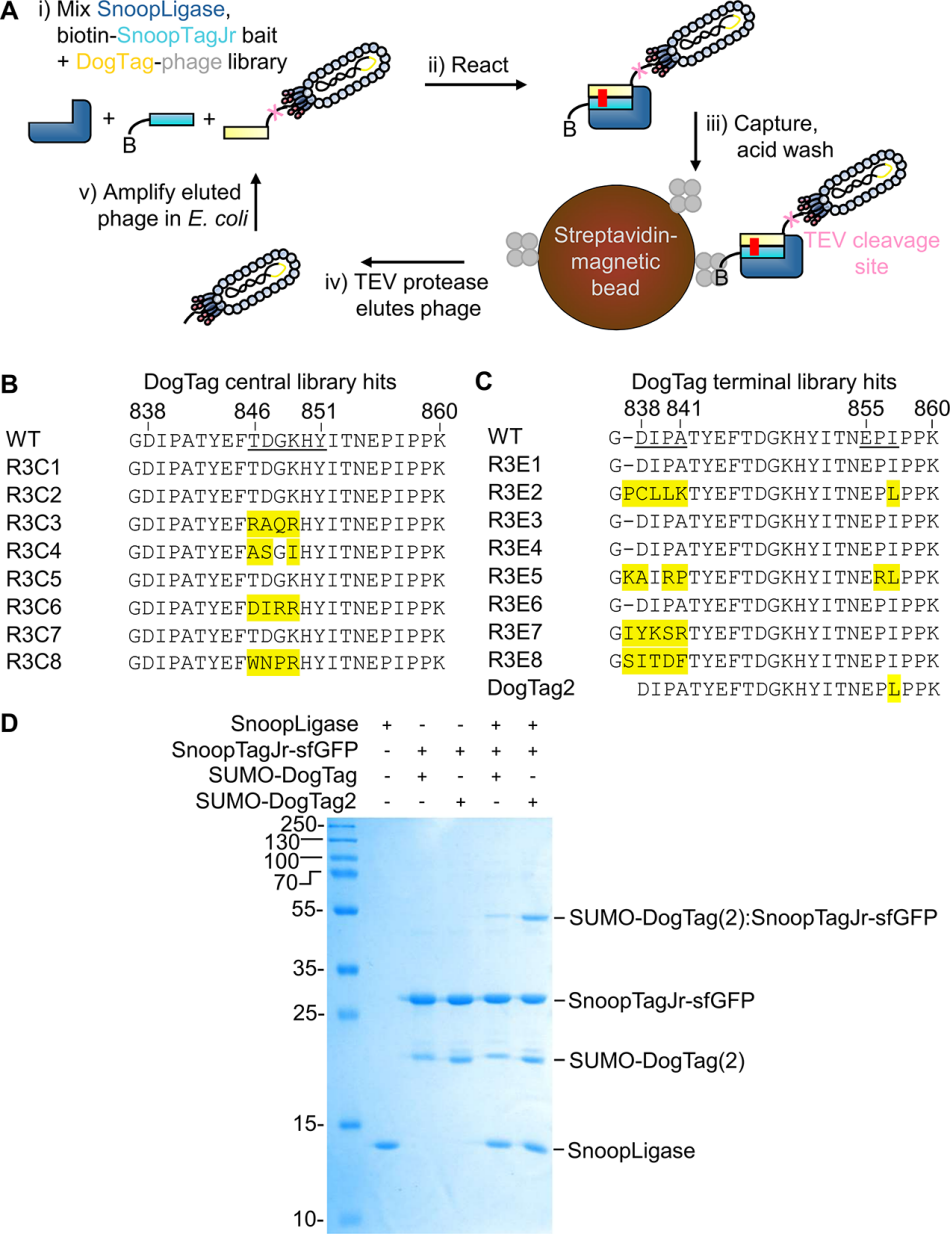
Directed evolution of DogTag2. (A) Cartoon of phage display selection of a faster DogTag. Biotinylated AviTag-SnoopTagJr-AffiHER2 was used as bait with reaction requiring SnoopLigase. B represents biotin, and the small gray circles are streptavidin. The TEV cleavage site is shown in pink and the isopeptide bond as a red line. (B) Selected amino acid sequences of DogTag clones from the central library. Residues underlined on WT were mutated within the library, with observed changes in yellow. R refers to the selection round; C and E are randomization routes. (C) Selected amino acid sequences of DogTag clones from the terminal library. (D) DogTag2 enhanced peptide–peptide ligation. SUMO-DogTag or SUMO-DogTag2 was ligated to SnoopTagJr-sfGFP using SnoopLigase (25 μM each) for 3 h at 21 °C in Tris-borate pH 7.4 (SDS–PAGE with Coomassie staining).

Building on previous experience on point mutations and library selections of RrgA,^10,14,15^ in parallel to the above libraries focusing on SnoopTagJr, we generated two focused libraries of DogTag, displayed as a pIII fusion on the M13 phage. In one library, we randomized residues 846–851 at the center of DogTag. In the other library, we randomized residues at each end of DogTag (838–841 and 855–857) (Figure 2B,C).

Initial selections with libraries of DogTag for reaction with biotinylated DogCatcher bait (following Figure 1C) quickly collapsed to wild-type (WT) DogTag after one or two rounds. Development of the previous ligation systems SnoopTag/SnoopCatcher and SnoopLigase involved incorporation of three mutations to the parent protein to create DogTag; these mutations, to favor the formation of β-hairpin conformation by DogTag, have already resulted in an order of magnitude improvement in reactivity.^10,14^ Hence, we hypothesized that WT DogTag was close to optimal for reaction with DogCatcher. Therefore, we changed to selecting DogTag variants for faster SnoopLigase-mediated coupling (Figure 2A). SnoopLigase has poor reactivity in NaCl-containing buffers, such as phosphate-buffered saline (PBS).^10^ Therefore, we conducted selections in this challenging buffer, along with 0.05% (v/v) TWEEN 20 to reduce non-specific binding. We employed a site specifically biotinylated bait of an affibody against the growth factor receptor HER2^32^ linked to SnoopTagJr (biotin-AffiHER2-SnoopTagJr). Again, the acid wash was designed to wash away non-covalent complexes. TEV protease allowed specific release of reactive phage from the beads (Figure 2A). After three rounds of selection of increasing stringency (from 1 μM bait in the first round to 100 nM bait in the third round), clones were picked, and representative sequences are shown in Figure 2B,C. The key pattern was strong selection for the original DogTag sequence, despite cloning the library into a vector based on the non-reactive DogTag N854A mutant, to minimize the abundance of parental WT DogTag sequence. These data suggest that DogTag is nearly optimal for SnoopLigase reaction. For the mutations we did see, there was little consensus except for DogTag I857L (Figure 2B,C). After evaluating a range of candidate DogTag mutants arising from the screen for SnoopLigase reaction speed, we proceeded with the I857L mutant. DogTag I857L was termed DogTag2 (Figure S2) and substantially enhanced SnoopLigase-directed reaction with SnoopTagJr compared to the parental DogTag (Figure 2D).

### Optimization of SnoopLigase Reactivity

**Figure 3:**
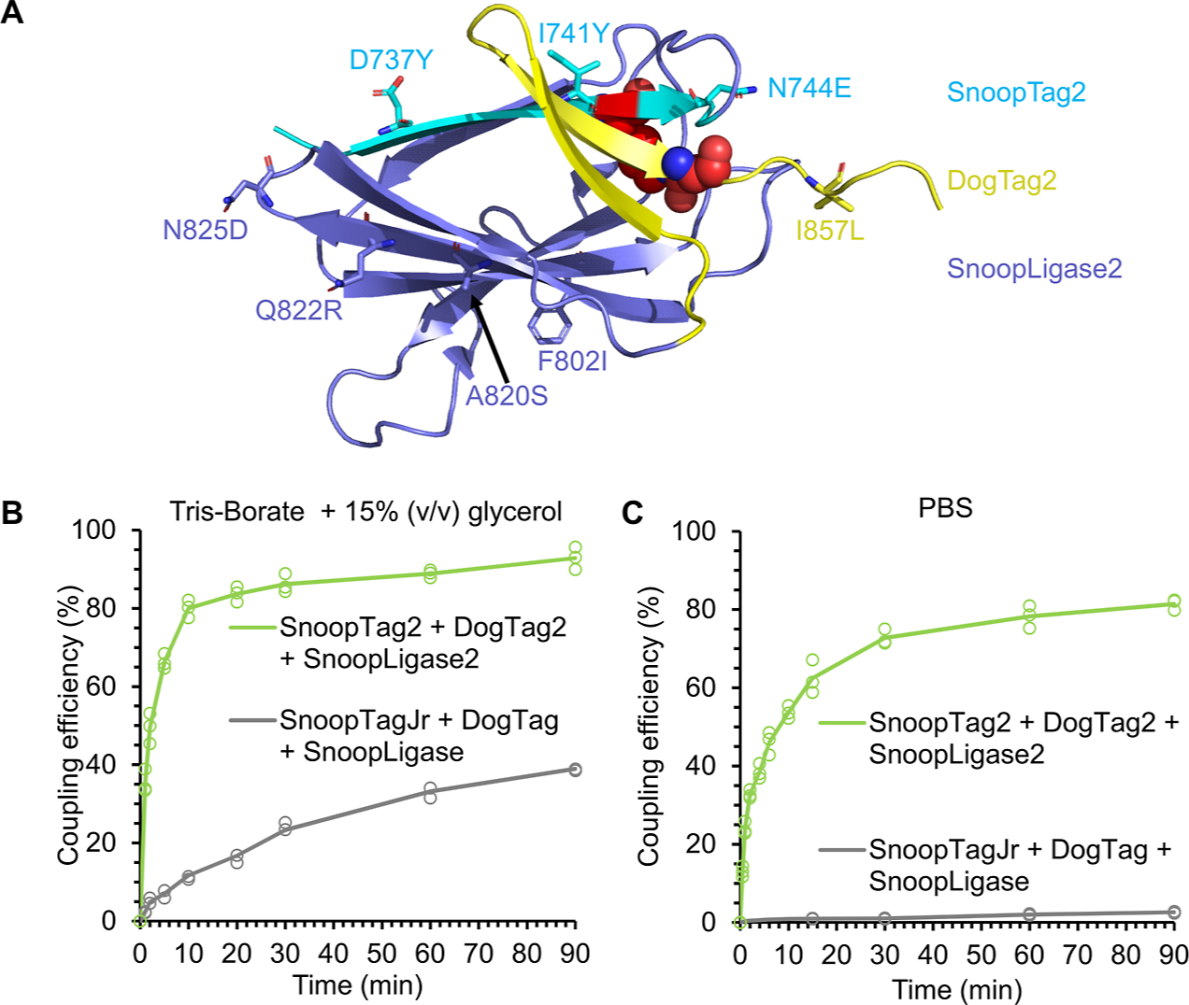
Properties of an enhanced SnoopLigase. (A) Location of new mutated residues in SnoopLigase2 (dark blue), SnoopTag2 (cyan), and DogTag2 (yellow) (schematic based on the parent domain, PDB 2WW8). (B) SnoopLigase2 enhances reaction in Tris-borate. SnoopTagJr- or SnoopTag2-sfGFP, SUMO-DogTag or -DogTag2, and SnoopLigase or SnoopLigase2 (25 μM each) were incubated for varying times at 21 °C in Tris-borate pH 7.4 with 15% (v/v) glycerol, before SDS–PAGE/Coomassie. Circles show each of the triplicate data points. (C) SnoopLigase2 enables rapid reaction in PBS. As in (B) in PBS pH 7.4. Some data points are overlapping.

DogCatcher and SnoopLigase are alternative ways of splitting the RrgA domain 4 (Figures S1 and 1B). One of the mutations that aided the production of the original SnoopLigase (A808P), to stabilize a β-turn, was involved in the generation of DogCatcher from the parental RrgA.^10,15^ Thus, we hypothesized that mutations identified by phage display to improve DogCatcher reactivity may also improve SnoopLigase reactivity. Incorporation of the best combination of these mutations from DogCatcher (F802I + A820S + Q822R + N825D) resulted in SnoopLigase2. F802I, A820S, and Q822R arose from a previous Catcher-based phage display library, selected for faster reaction with DogTag.^15^ N825D was previously designed to increase the surface polarity and so potentially enhance the solubility.^15^ These mutations are illustrated in Figure 3A, and the sequence is listed in Figure S2. SnoopLigase2 was expressed solubly at 8 mg/L shake flask culture in *Escherichia coli*. Our SnoopLigase2 stock was 260 μM, so the protein has good solubility. Formation of the expected SnoopTag2/DogTag2 ligation product by SnoopLigase2 was validated by electrospray ionization mass spectrometry (Figure S3).

Bringing together these improvements in each component of the SnoopLigase system, we carefully characterized the change in overall performance. The original SnoopLigase reaction was sensitive to buffer conditions, preferring Tris-borate with 15% (v/v) glycerol and minimal NaCl.^10^ Under these optimized conditions, the original set (SnoopTagJr, DogTag, and SnoopLigase) performed competently (∼40% coupling after 90 min), but the new set (SnoopTag2, DogTag2, and SnoopLigase2) was much more efficient, giving ∼80% coupling in only 10 min (Figure 3B). When moving to the common buffer for molecular biology of PBS at pH 7.4, the original set had very little reactivity (<5% after 90 min) but the new set reacted efficiently (Figure 3C). Since it was hard to get a large amount of coupling with the original set in PBS, we quantified initial rates based on the time to 10% coupling. The new set accelerated the reaction 11-fold in Tris-borate/glycerol buffer and 1300-fold in PBS (Figure S4).

To determine the role of each modified tag in the improved performance, we then tested SnoopLigase2 with each new or original tag. Both SnoopTag2 and DogTag2 contributed substantially to the coupling efficiency (Figure S5).

### SnoopLigase2 Is Efficient in a Diverse Range of Conditions

**Figure 4:**
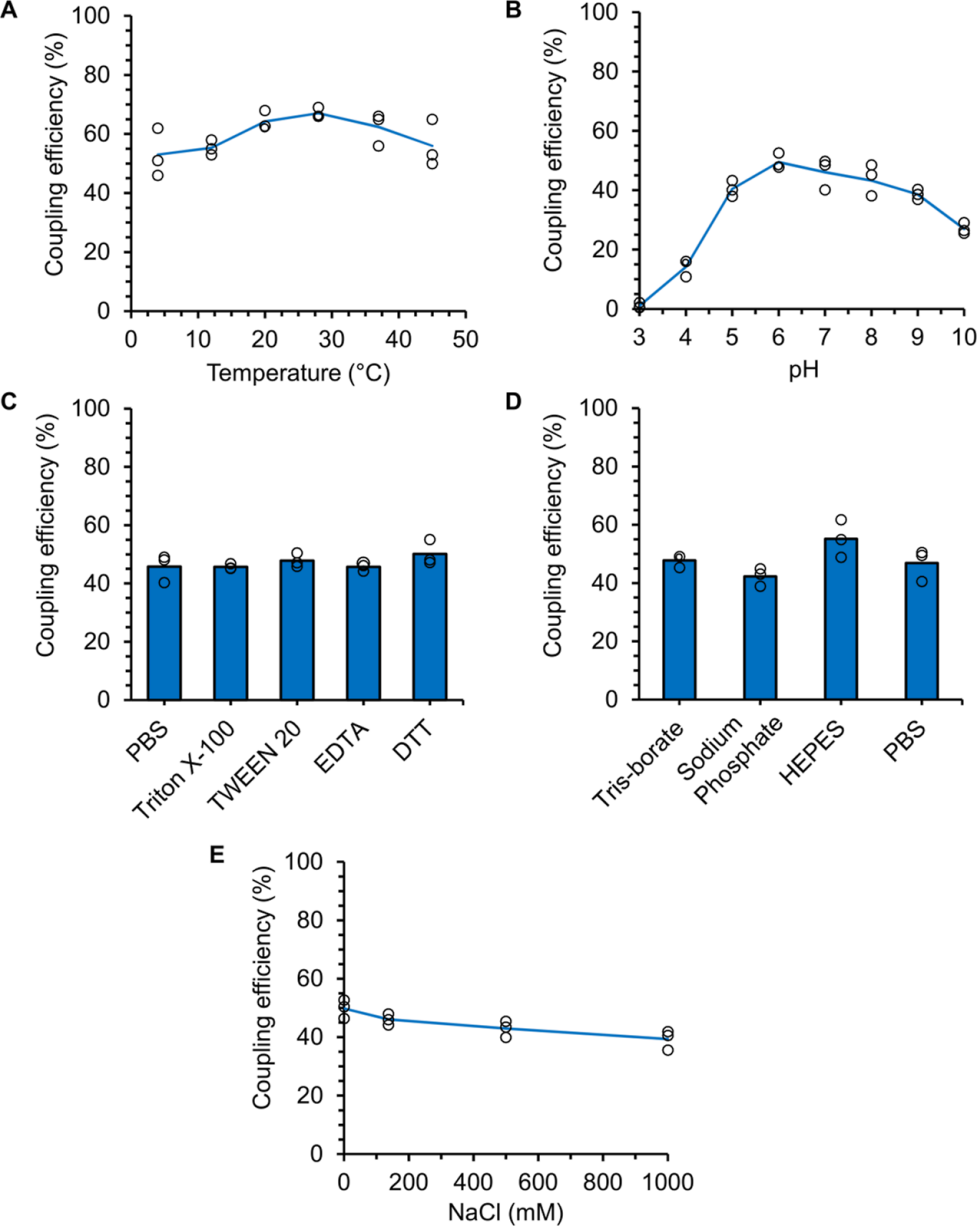
SnoopLigase2 reacts over a range of conditions. A short time was chosen so that the extent of reaction would be sensitive to effects on reaction rate. (A) Temperature dependence of SnoopLigase2 reaction. SnoopLigase2 ligated SnoopTag2-sfGFP and SUMO-DogTag2 (20 μM each) in HEPES pH 7.5 for 10 min at the indicated temperature. (B) pH dependence. As in (A) in succinate–phosphate–glycine at the indicated pH for 9 min at 25 °C. (C) Additive compatibility. As in (A) in PBS pH 7.4 for 8 min at 25 °C with 1% (v/v) Triton X-100, 1% (v/v) TWEEN 20, 1 mM EDTA, or 2 mM DTT. (D) Buffer compatibility. As in (A) for 12 min at 25 °C with the indicated buffer. (E) Salt dependence. As in (A) in HEPES pH 7.5 for 12 min at 25 °C with the indicated additional NaCl. Circles show each of the triplicate data points, with the line or bar indicating the mean.

After demonstrating that SnoopLigase2 is highly reactive in PBS, we tested SnoopLigase2 ligation of SnoopTag2 and DogTag2 in a wide range of conditions. We analyzed reactions at a short time point that helped us to detect increased or decreased efficiency compared to the control reaction. SnoopLigase2 ligated efficiently at all tested temperatures (4–45 °C) (Figure 4A). SnoopLigase2 reaction was active over an unusually broad range of pH values, with some reaction even at pH 4 or 10 (Figure 4B). We then tested widely used buffer additives. SnoopLigase2 reactivity was unaffected by the commonly used detergents Triton X-100 or TWEEN 20 (Figure 4C). Addition of ethylenediaminetetraacetic acid (EDTA) (a commonly used metal ion chelator) had no effect on SnoopLigase2 reaction (Figure 4C). There are no cysteines in SnoopTag2, DogTag2 or SnoopLigase2, so, naturally, the reducing agent dithiothreitol (DTT) had no effect on the reaction (Figure 4C). Despite the sensitive buffer dependence of the original SnoopLigase,^10^ we found little effect on SnoopLigase2 ligation when comparing Tris-borate, sodium phosphate, HEPES, or PBS, all at pH 7.5 (Figure 4D). SnoopLigase reactivity is decreased in the presence of NaCl.^10^ SnoopLigase2 reactivity was maintained in all concentrations up to 1 M NaCl (Figure 4E). Overall, the new set provides a system for peptide–peptide ligation that is highly robust to reaction conditions.

We demonstrated that both SnoopTag2 and DogTag2 perform highly efficient ligation with each tag present at either the N- or C-terminus of TG2. Ligation of AviTag-DogTag2-MBP (MBP = maltose-binding protein) to TG2x bearing N- or C-terminal SnoopTag2 depleted 97–98% of the TG2x substrate (Figure S6A). Ligation of SnoopTag2-sfGFP to TG2x bearing N- or C-terminal DogTag2 depleted ∼100% of TG2x substrate (Figure S6B). Efficient ligation by SnoopLigase2 is also possible with DogTag2 at an internal site, in a loop of sfGFP,^15^ although an excess of one substrate is required for efficient reaction of the other substrate (Figure S7).

### Reaction of SnoopLigase2 in Human Cells

**Figure 5:**
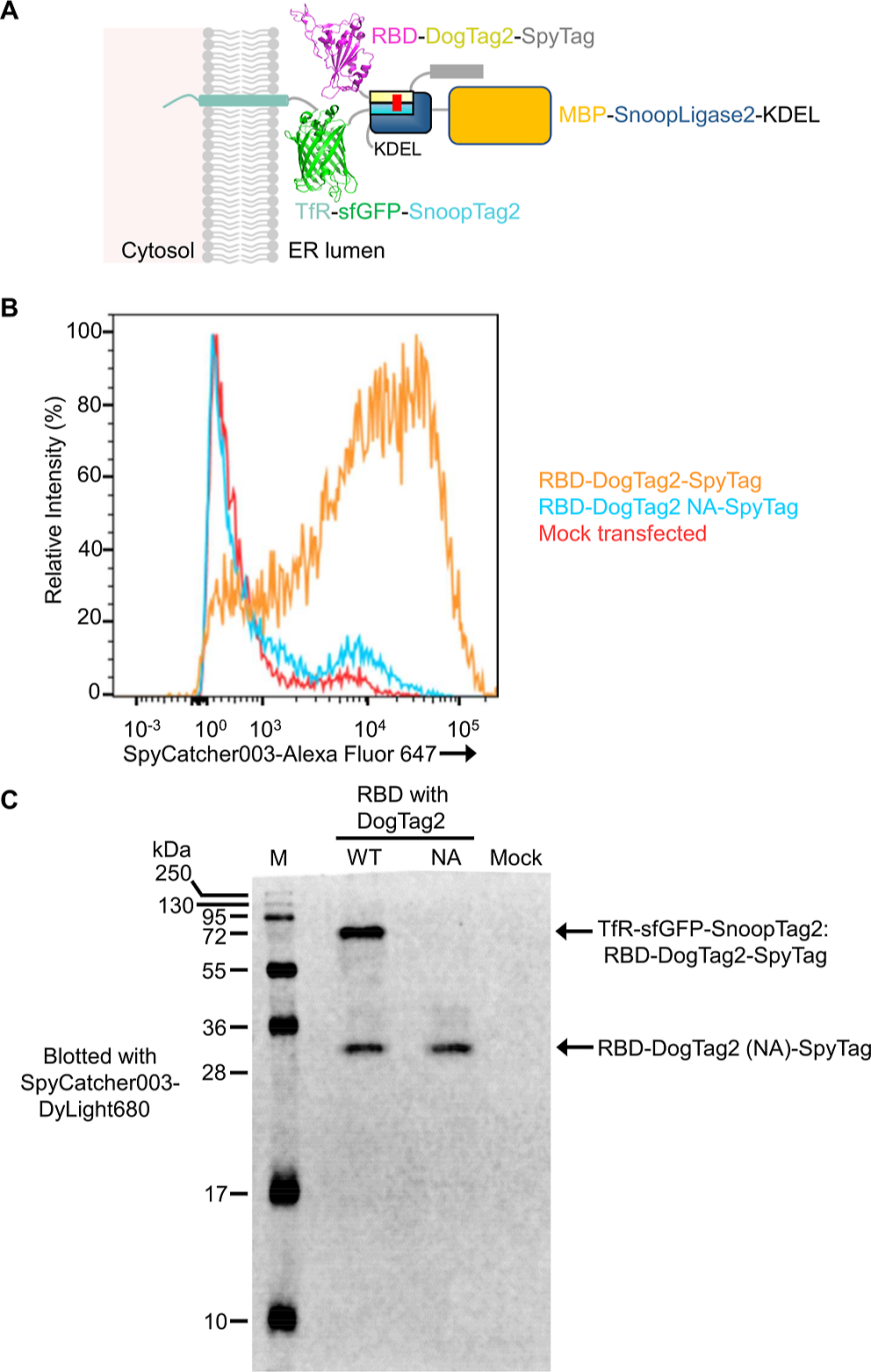
SnoopLigase2 ligates in human cells. (A) Schematic of constructs for cellular reaction. The cytosolic tail and transmembrane helix of TfR are genetically fused to sfGFP and SnoopTag2. RBD is fused to DogTag2 and SpyTag. SnoopLigase2 ligates SnoopTag2 to DogTag2 via an isopeptide bond (red line). Constructs may later traffic from the endoplasmic reticulum to the plasma membrane. (B) SnoopLigase2 allows covalent display at the surface of Expi293F cells. The SpyTag linked to RBD-DogTag2 was detected using SpyCatcher003-Alexa Fluor 647 by flow cytometry. The constructs with the unreactive DogTag2 NA or mock-transfected cells were negative controls. (C) Western blotting of SnoopLigase2 ligation. Post-nuclear supernatant from Expi293F cells, transfected as in (B), was analyzed by western blotting. SpyTag on the construct was detected by near-infrared fluorescence imaging with SpyCatcher003-DyLight680.

SnoopLigase reaction was previously only shown on isolated proteins.^10^ SnoopLigase2 reaction tolerance was markedly improved over SnoopLigase, notably being highly active in the presence of different buffers and without the need for glycerol. Therefore, we tested whether SnoopLigase2 would be able to react with SnoopTag2 and DogTag2 constructs in human cells. We previously demonstrated efficient surface display of SpyCatcher003 at the plasma membrane of Expi293F cells, directing export with an N-terminal transferrin receptor (TfR) transmembrane helix linked to sfGFP to quantify total protein levels.^19^ We modified the construct to display SnoopTag2, generating TfR-sfGFP-SnoopTag2 (Figure 5A). SnoopLigase2 was expressed from a second plasmid with a signal sequence for secretion, fused to MBP for high solubility, and bearing a C-terminal KDEL sequence (MBP-SnoopLigase2-KDEL) to favor endoplasmic reticulum retention. SnoopLigase2 here included the N775Q mutation to remove a potential N-linked glycosylation site. DogTag2 was expressed from a third plasmid as a fusion to the receptor-binding domain (RBD) from SARS-CoV-2. SpyTag was present at the C-terminus to help detection (giving RBD-DogTag2-SpyTag) (Figure 5A). Without SnoopLigase2 activity, RBD-DogTag2-SpyTag should be secreted to the medium. SnoopLigase2 ligation would allow RBD-DogTag2-SpyTag to be anchored covalently to the surface of the expressing cells, leading to staining with fluorescent SpyCatcher003. We also cloned RBD-DogTag2 NA-SpyTag, where mutation of DogTag2’s reactive Asn to Ala prevents covalent bond formation to SnoopTag2. Co-transfection of Expi293F cells with the plasmids encoding the SnoopLigase2, SnoopTag2, and DogTag2 constructs indeed allowed high-level surface staining with SpyCatcher003-Alexa Fluor 647 (Figure 5B). The unreactive DogTag2 NA variant gave negligible cell staining with SpyCatcher003-Alexa Fluor 647, similar to the level of mock-transfected cells (Figure 5B). Therefore, SnoopLigase2 activity enabled stable anchoring of RBD-DogTag2-SpyTag at the surface of human cells.

To validate covalent bond formation, we performed western blotting in this system. SpyTag-bearing proteins in the cell lysate were detected on the membrane by near-infrared fluorescent imaging using SpyCatcher003-DyLight680 (Figure 5C).^19^ Only in the case of cells expressing SnoopLigase2, SnoopTag2 and DogTag2 was ligation detected. No bands were seen for mock-transfected cells, consistent with the previously identified specificity of SpyCatcher003 reaction.^19^ Free RBD-DogTag2 (WT/NA)-SpyTag was also detected in the cell lysate (Figure 5C), which may not yet have had time to react. There was only one new product from SnoopLigase2 ligation (Figure 5C), indicating that SnoopLigase2 did not detectably ligate DogTag2 to other cellular proteins. Overall, SnoopLigase2 allows efficient and selective peptide–peptide ligation in the mammalian secretory pathway.

### SnoopLigase2 Performs Efficient Modular Ligation of TG2 to Cargo

**Figure 6:**
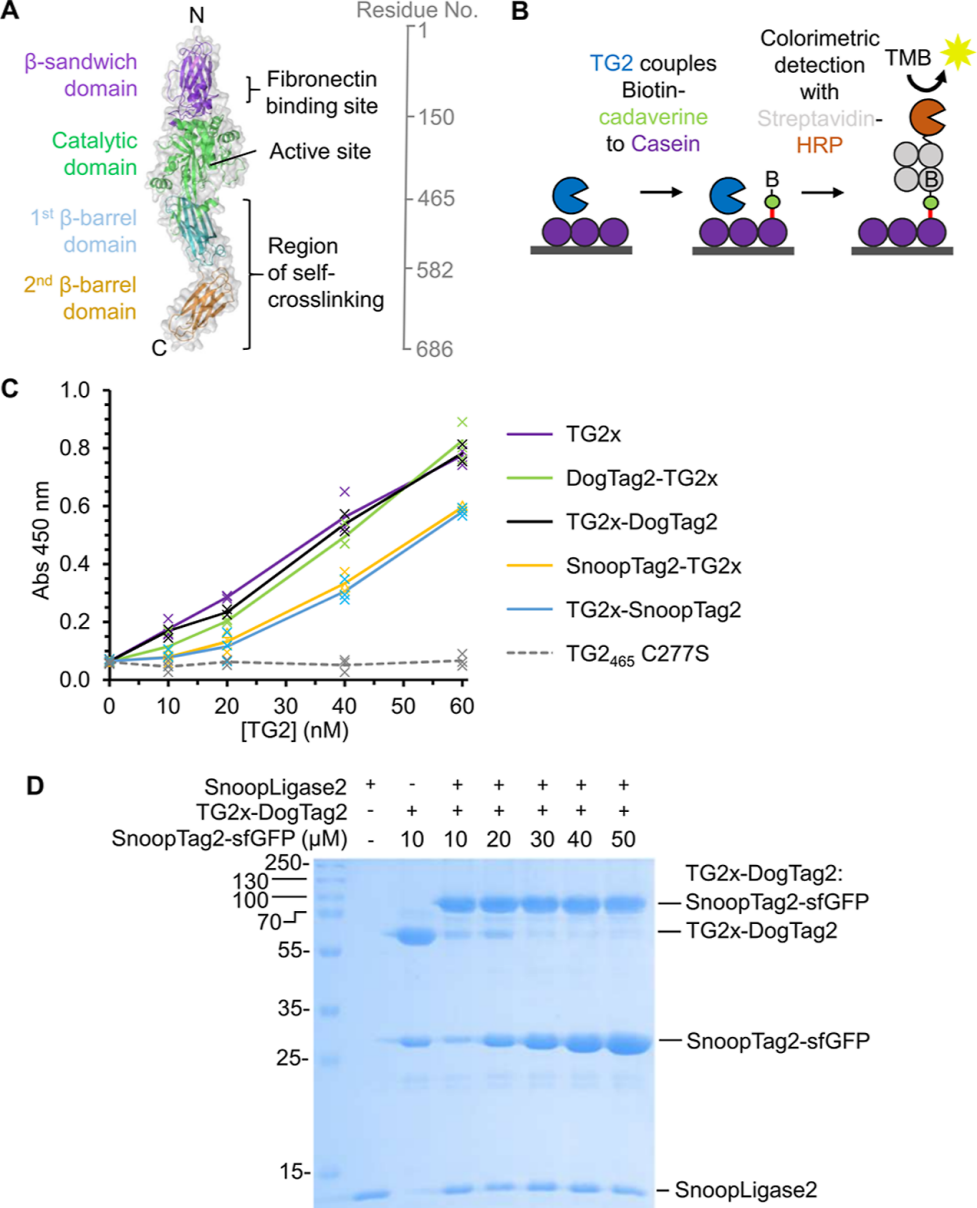
Ligation of cargo to modified TG2. (A) Crystal structure of TG2 (PDB 2Q3Z), including key features of interest and amino acid residue numbers for each domain. (B) Schematic of the colorimetric assay for transamidase activity of TG2. B represents biotin. (C) Cadaverine assay of modified TG2 with DogTag2 or SnoopTag2 fused to either N- or C- terminus. Assays were performed for 30 min at 37 °C with varying concentration of the indicated TG2 variant. Crosses show each of the triplicate data points, with the line indicating the mean. (D) Coupling of TG2x-DogTag2 to SnoopTag2-sfGFP by SnoopLigase2. SnoopTag2-sfGFP was incubated with 10 μM of TG2x-DogTag2 and 20 μM SnoopLigase2 in HEPES-buffered saline pH 7.4 for 16 h at 4 °C and then analyzed by SDS–PAGE in reducing conditions with Coomassie staining.

TG2 is a multi-domain enzyme which catalyzes acyl transfer between Gln donors and amine acyl acceptors (Figure 6A).^22^ TG2 is also a tool with potential for medical application and synthetic biology, given its ability to direct covalent reaction with a range of cell surface and ECM proteins, ligating amine donors or itself.^21,26^ Building on previous TG2 modification, we expressed a truncated form of TG2, containing residues 1–465 (Figure 6A), to decrease TG2’s self-reactivity.^33^ We also included the C230S mutation to improve resistance to oxidative inactivation.^34^ We named the TG2_465_ C230S variant as TG2x. While cargo proteins could potentially be genetically fused to TG2x for tagging to endogenous proteins, fusion restricts applications to cargo that can be expressed in active form in *E. coli*. Therefore, we used SnoopLigase2 to facilitate modular ligation of previously expressed cargo to TG2. First, we genetically fused SnoopTag2 or DogTag2 at the N- or C-terminus of TG2x. In all cases, TG2x was expressed in soluble form at a yield of ∼2 mg/L culture. The activity of TG2 in transamidation can be measured by detecting the incorporation of an amine (biotin cadaverine) into an immobilized Gln donor (casein) using a plate-based assay^35^ (Figure 6B). C277S is a negative control, removing the key reactive Cys at the TG2 active site^36^ (Figure 6C). We found that transamidase activity was retained for all tagged-TG2 constructs, with little change upon DogTag fusion but some decrease in activity with SnoopTag2 fusion (Figure 6C). It may be that one or more of the 3 Lys present in SnoopTag2 could react with TG2. This side reaction would couple SnoopTag2 to casein rather than coupling biotin cadaverine, resulting in a lower signal. Overall, we selected TG2x-DogTag2 as our platform for modular ligation, showing good soluble expression and high enzymatic activity.

Next, we tested the use of SnoopLigase2 for ligation to TG2 fusions. We incubated TG2x-DogTag2, SnoopLigase2, and SnoopTag2-sfGFP as a model partner for 16 h at 4 °C. A 3-fold excess of SnoopTag2-sfGFP over TG2x-DogTag2 resulted in >99% coupling of TG2x-DogTag2 (Figures 6D and S8A). This high yield facilitated separation of SnoopLigase2 and substrates from the ligated product by size exclusion chromatography (Figure S8B). The purified ligated product was assayed for transamidase activity and found to be active (Figure S9). The decrease in signal may relate to sfGFP being a substrate for TG2, which results in some transamidation of sfGFP Lys residues.

### SnoopLigase2 Is Hard to Release from Ligated SnoopTag2:DogTag2

Reaction of SnoopTag2 with DogTag2 by SnoopLigase2 generates a tripartite complex similar to the parental RrgA domain 4 (Figure 1B). For the parent SnoopLigase, we found that this complex with its covalently linked SnoopTagJr and DogTag substrates is stably assembled.^10^ However, the ligated SnoopTag:DogTag could be released by low pH or high concentrations of imidazole.^10^ To investigate the stability of the complex here, we generated a fusion of SnoopLigase2 with HaloTag for covalent ligation to HaloLink resin.^37^ HaloTag-SnoopLigase2 efficiently ligated SnoopTag2-sfGFP to TG2x-DogTag2 (Figure S10A). We then attempted to elute the ligated product from the resin using 3 M imidazole or pH 2.5 buffer or competing the SnoopTagJr:DogTag peptide. However, the interaction of SnoopLigase2 with the ligated product survived each of these harsh conditions (Figure S10B). We were able to free the ligated SnoopTag2-sfGFP:TG2x-DogTag2 from HaloTag-SnoopLigase2 only upon boiling in sodium dodecyl sulfate (SDS) (Figure S10B). Therefore, the evolution of the new components has selected for a high stability heterotrimer, and so SnoopLigase2 is acting as a single-turnover catalyst.

### TG2 Fusion Allows Modular and Stable Growth Factor Anchoring

We next applied SnoopLigase2 to ligate TG2 with TGFα. TGFα has a multitude of physiological effects, including cell proliferation, development, and wound healing.^27^ Like many growth factors, the TGFα activity depends on the sites of release as well as on the sites of anchoring.^27,38^ Linkage to TG2, which interacts with cell surface and extracellular matrix components,^24^ may demonstrate modular enhancement of the stability of TGFα anchoring in the extracellular space. We ligated our TG2x-DogTag2 construct to SnoopTag2-TGFα variants, with 98% reaction of TG2x-DogTag2 (Figure S11A). SnoopTag2-TGFα had been expressed in *E. coli* by refolding from inclusion bodies (Figure S11B). The ligated TG2-DogTag2:SnoopTag2-TGFα product was isolated by size exclusion chromatography (Figure S11C). In contrast, when we cloned a direct genetic fusion of TG2x-TGFα from our expression and refolding, we could only obtain heterogeneous aggregates (Figure S11D,E), supporting the value of modular conjugation to TG2.

**Figure 7:**
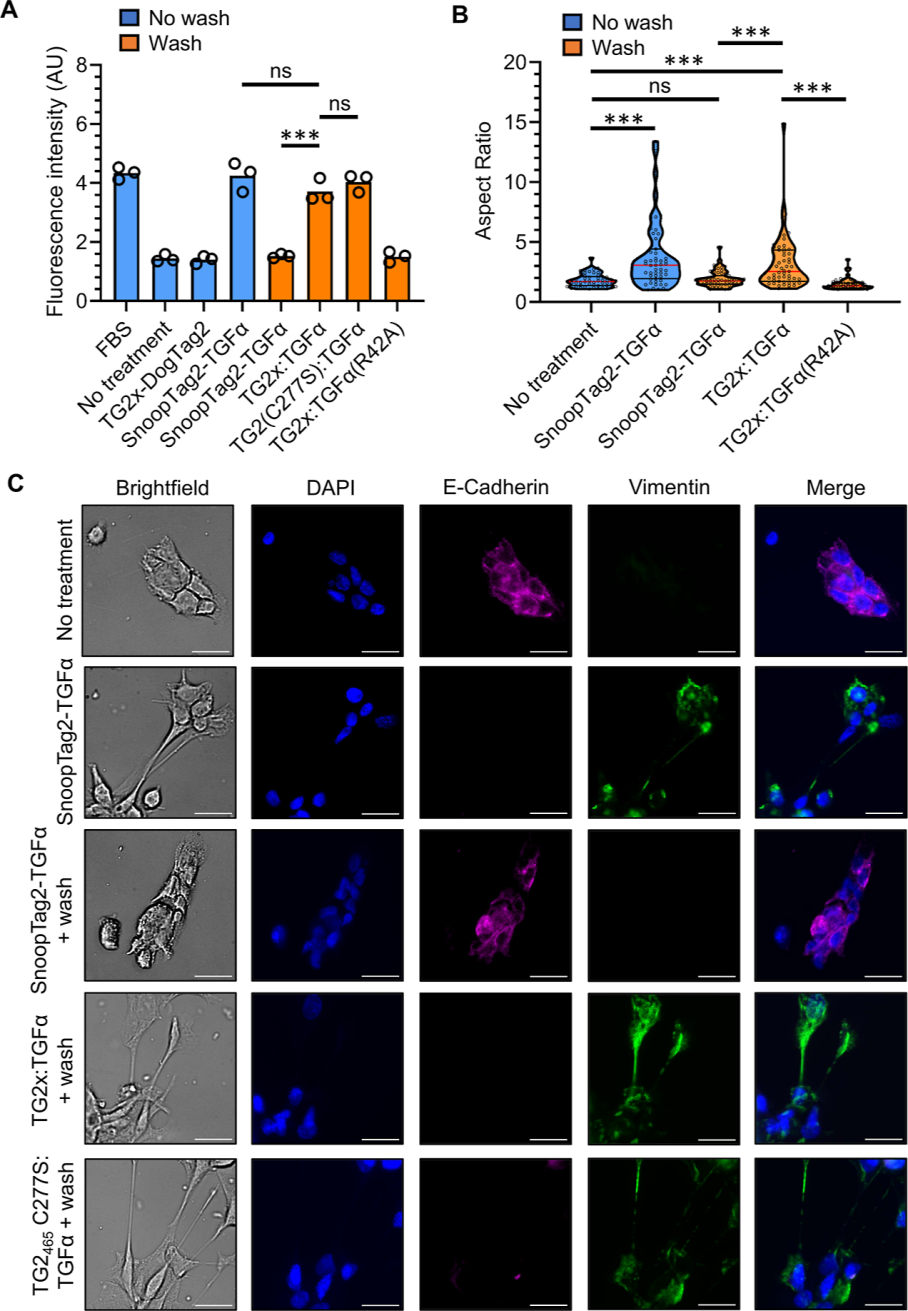
TGFα activity is enhanced by TG2-mediated anchoring to the cell surface. (A) TG2 anchoring enhanced TGFα-induced proliferation. DU145 cells were incubated with FBS or the indicated protein. Samples in orange were washed with media after 30 min, while blue samples were not washed. All samples were then incubated for 3 days, before proliferation was measured by resazurin. Circles show each triplicate data point, with the bar indicating the mean. Selected pairs were compared using an unpaired two-tailed Student’s *t*-test (*n* = 3, ***p < 0.001). AU = arbitrary units. (B) TG2 anchoring increased TGFα-induced change in cell morphology. DU145 cells were incubated as in (A). Morphology was quantified by fluorescence microscopy. Each cell is marked as a black circle. The distribution is marked as a violin plot. The red line denotes the median and the black lines denote the interquartile range. Selected pairs were compared using an unpaired two-tailed Welch’s *t*-test (*n* = 51, ***p < 0.001). (C) TG2 anchoring enhanced TGFα-induced epithelial–mesenchymal transition detected by fluorescence microscopy. Cells were treated with the indicated proteins as in (A), with or without washing to remove any unanchored growth factor. Cells are shown in brightfield (grayscale) and after staining with DAPI (blue) or antibodies to E-cadherin (magenta) and vimentin (green). The right column shows a merge of DAPI (blue), E-cadherin (magenta), and vimentin (green) images. Scale bar = 15 μm.

We tested the stability of the interaction of TG2x-DogTag2:SnoopTag2-TGFα with the cell surface by incubation with the DU145 prostate cancer cell line and then subjecting the cells to washes. To determine the ability of TGFα to maintain its effect on cell proliferation, we performed a resazurin assay. SnoopTag2-TGFα increased cell proliferation of DU145 cells to levels comparable to treatment with 10% (v/v) fetal bovine serum (FBS) (Figure 7A). However, when cells were washed after addition of SnoopTag2-TGFα, cell proliferation was no higher than cells incubated in RPMI alone. Cells treated with TG2x-DogTag2:SnoopTag2-TGFα showed significantly greater cell proliferation after washing than cells treated with SnoopTag2-TGFα (*p* < 0.001, *n* = 3) (Figure 7A). Furthermore, proliferation of cells treated with TG2x-DogTag2:SnoopTag2-TGFα and washed was equivalent to proliferation of cells treated with SnoopTag2-TGFα without washes (ns, *n* = 3). This increase in cell proliferation is not a direct effect of TG2 because TG2x-DogTag2 alone did not cause an increase. TGFα(R42A) displays impaired activation of EGFR.^39^ TG2x-DogTag2:SnoopTag2-TGFα(R42A) had no effect on cell proliferation (Figure 7A), indicating that the observed increase in cell proliferation by TG2x-DogTag2:SnoopTag2-TGFα is caused by prolonged TGFα activity. The product TG2_465_ C277S-DogTag2:SnoopTag2-TGFα is devoid of transamidase activity and displays equivalent performance to TG2x-DogTag2:SnoopTag2-TGFα (ns, *n* = 3) (Figure 7A). These findings suggest that TG2-mediated cell surface retention results from non-covalent interactions by TG2. TG2 forms well-studied interactions with fibronectin,^40^ integrins,^41^ and heparan sulfate proteoglycans,^42^ which may be driving the observed affinity for the cell surface. TG2(1–465) previously showed very high affinity (*K*_d_ 0.3 nM) for a fibronectin fragment.^40^ TG2’s interaction with fibronectin leads to an RGD-independent adhesion to cells, which depends on heparan sulfate.^41^ In that study, it was also found that the TG2 catalytic activity was not required for strong interaction of TG2 with the cells.^41^

TGFα has been shown to induce epithelial-to-mesenchymal transition (EMT) in DU145 cells, which normally show epithelial morphology.^43^ EMT is a major transition in cell behavior that occurs during embryonic development, wound healing, and cancer metastasis.^44^ Cells modify their adhesion molecules, migratory capacity, and extracellular matrix secretion.^44^ DU145 cells were incubated with various TGFα constructs and then stained with the actin-binding ligand phalloidin by fluorescence microscopy to allow determination of cell shape. TGFα activity promoted a morphological change from rounded to spindle-shaped cells, which we quantified by the cell aspect ratio (Figure 7B). Cells incubated in RPMI media formed colonies of rounded cells with a median aspect ratio of 1.7. Cells treated with SnoopTag2-TGFα formed elongated spindle shapes with a significantly larger median aspect ratio of 3.1 (*p* < 0.001, *n* = 51), indicative of EMT. This effect was also observed when cells were washed after addition of TG2x-DogTag2:SnoopTag2-TGFα (*p* < 0.001, *n* = 51). In contrast, treatment with SnoopTag2-TGFα followed by washes was not significantly better at inducing EMT than the no treatment condition (ns, *n* = 51) (Figure 7B).

We further analyzed TGFα-induced effects by immunostaining DU145 cells for established markers of EMT—the adheren junction component E-cadherin and the intermediate filament protein vimentin^44^ (Figure 7C). DU145 cells incubated in RPMI expressed high levels of E-cadherin at cell–cell junctions and did not express detectable levels of vimentin. Treatment with SnoopTag2-TGFα resulted in downregulation of E-cadherin and upregulation of vimentin, consistent with the cells undergoing EMT^43,44^ (Figure 7C). Washing cells after addition of SnoopTag2-TGFα resulted in no changes in vimentin and E-cadherin expression, whereas cells treated with TG2x-DogTag2:SnoopTag2-TGFα displayed upregulated vimentin and downregulated E-cadherin (Figure 7C). Overall, these results indicate that SnoopLigase2-mediated ligation of TG2 to TGFα enables retention of both TG2 enzymatic activity and TGFα cellular activation. By combining these two components, we have enabled TG2 to anchor TGFα stably in the extracellular environment, leading to proliferation, cell elongation, and EMT.

## Discussion

We have established a peptide–peptide ligation system comprising SnoopLigase2, SnoopTag2, and DogTag2 through a combination of directed evolution and rational design. Coupling goes to >90% completion in ∼90 min under conditions where the peptide ligases SnoopLigase^10^ and SpyLigase^17^ take >24 h to react. Furthermore, SnoopLigase2 reacts rapidly in buffers containing high NaCl, detergent, and other commonly used buffer components. This general applicability is in marked contrast to the original SnoopLigase that requires a specialized buffer for efficient reaction.^10^ SnoopLigase2 reactions had a greater tolerance to variations in temperature than SpyTag/SpyCatcher, SnoopTag/SnoopCatcher, or DogTag/DogCatcher systems,^14,15,19^ with little change in reactivity observed even from 4 to 45 °C. The pH tolerance of SnoopLigase reactions was also unusually broad (pH 4–10). DogTag/DogCatcher reacts slowly below pH 7,^14,15^ while SpyTag003/SpyCatcher003 reacts slowly above pH 8.5.^19^ In future work, this pH tolerance may allow SnoopLigase2 coupling even in the acidic compartments of the cell, such as secretory granules and the endolysosomal system.^45^ Together with the tolerance of the reaction to salt, temperature, and redox, this explains why SnoopLigase2 can couple proteins in mammalian cells for display on the plasma membrane. SnoopTag2 or DogTag2 reacts efficiently when fused to the N- or C-terminus of substrates. Alternative technologies such as butelase, subtiligase, sortases, and inteins have been validated in mild conditions (pH 7–9, 4–37 °C).^6−8,46^ Here, we have demonstrated highly efficient coupling at pH and temperatures outside this range and thus extended the current capabilities of the protein–protein conjugation toolbox. In particular, SnoopLigase2-mediated coupling at extreme pH may find application in attaching proteins to surfaces on array chips under conditions that disfavor non-specific binding.

The stable association of SnoopLigase2 with SnoopTag2:DogTag2 is not surprising in light of our selection approach, which did not enforce multiple turnovers. However, in previous applications of SnoopLigase for enzyme resilience and vaccine development, SnoopLigase remaining bound did not impair functional activity of the ligated partners.^10,11^ There are a range of ways to trimerize proteins, but homotrimers are much more common than heterotrimers.^47−49^ In addition, coiled-coil-based heteromerization units can have impaired solubility when expressed in the absence of their cognate partners and often have unintended homodimerization.^47,50^ Therefore, the SnoopLigase2 system may find application in the general challenge of assembling three separately expressed components into a precise and stable complex.

Modularity is a highly desirable feature of emerging protein biotechnologies, significantly enhancing the range of applications and simplifying use.^51^ This simplicity then greatly increases the likelihood of a given technology realizing its potential and being utilized by the research community. Here, we have demonstrated how SnoopLigase2 can be applied to introduce modularity to other biotechnologies. TG2x is capable of high-affinity decoration of the extracellular space, but expression is limited to *E. coli,* and genetic fusion of cargo is challenging. Many proteins of therapeutic interest are reliant on post-translational modification for correct folding and activity, as well as often being insoluble when expressed in *E. coli*.^52^ By applying the SnoopLigase2 system to TG2x, cargo can be expressed, purified, and prepared as required before coupling to TG2x. This functionalization opens up a range of applications, such as anchoring of signaling effectors in wound repair, transplantation, and cancer immunotherapy.^38,53^ The high stability and intricate ordering of extracellular matrix components may allow future functionalization of biomaterials with modules for cellular signaling^21^ or controlled interaction with light (from structural color to transparency).^1,2^

## Experimental Procedures

### Bacterial Strains

Plasmids were amplified using *E. coli* NEB Turbo cells (New England Biolabs) or *E. coli* K12 TG-1 cells (Lucigen), which were grown in LB medium with an antibiotic at 37 °C. Proteins were expressed in *E. coli* BL21(DE3) RIPL (Agilent), *E. coli* C41(DE3) (a kind gift from Dr. Anthony Watts, University of Oxford), or *E. coli* T7 Express (DE3) (New England Biolabs) cells. Phage production for selections was carried out using *E. coli* K12 TG-1 cells grown in 2× TY medium.

### Cell Lines

Expi293F cells (Thermo Fisher) were maintained in Expi293 Expression media (Thermo Fisher) supplemented with 50 U/mL penicillin/streptomycin (Thermo Fisher). Cells were grown in a humidified Multitron Cell incubator (Infors HT) at 37 °C with 7% (v/v) CO_2_, rotating at 110–125 rpm. DU145 cells, a human prostate cancer cell line from Cancer Research UK Clare Hall Laboratories, were grown to confluence in RPMI 1640 (Gibco) + 10% (v/v) FBS (Gibco) + 1% (v/v) penicillin/streptomycin (Gibco) and maintained at 37 °C with 5% (v/v) CO_2_.

### Plasmids and Cloning

Site-directed mutagenesis and PCR-based cloning were carried out using Q5 High-Fidelity 2× master mix (New England Biolabs) and Gibson assembly. Residue numbering follows PDB 2WW8.^29^

Based on pBAD-DsbA(ss)-HA tag-SpyDock2.0 C49S-pIII (GenBank ON131078),^54^ we used Gibson assembly to generate pBAD-DsbA(ss)-HA tag-SnoopTagJr-TEVs-pIII (where TEVs is the cleavage site for TEV protease), pBAD-DsbA(ss)-HA tag-SnoopTagJr KA-TEVs-pIII, where the reactive K742 of SnoopTagJr is mutated to Ala, preventing isopeptide bond formation, pBAD-DsbA(ss)-HA tag-DogTag-TEVs-pIII, and pBAD-DsbA(ss)-HA tag-DogTag NA-TEVs-pIII, where the reactive N854 is mutated to Ala, preventing isopeptide bond formation. pET28a-SnoopTagJr-AffiHER2 was previously described.^10^ pET28a-AviTag-SnoopTagJr-AffiHER2 was derived from pET28a-SnoopTagJr-AffiHER2 by Gibson assembly. pET28a-SnoopTag2-AffiHER2 (GenBank OQ923247, Addgene 201801) was derived from pET28a-SnoopTagJr-AffiHER2 by Gibson assembly. pET28a AviTag-DogTag-MBP was described previously^15^ (GenBank MZ365293, Addgene 171773). pET28a AviTag-DogTag2-MBP (GenBank OQ923248, Addgene 201802) was derived from pET28a AviTag-DogTag-MBP by Gibson assembly. pET28a-AviTag-DogCatcher-MBP was previously described^15^ (GenBank MZ365308, Addgene 171928). pET28a SnoopLigase2 (GenBank OQ923250, Addgene 201803) was derived from pET28a SnoopLigase (GenBank MG867372), described in ref (10) by Gibson assembly to include the F802I, A820S, Q822R, and N825D mutations. pET28a Cys-SnoopCatcher N847D was derived from pET28a SnoopCatcher (GenBank KU500646, Addgene 72322)^14^ by introducing the N847D mutation and a Cys adjacent to the N-terminal His_6_ sequence by Gibson assembly. pENTR4 TfR-sfGFP-SnoopTag2 (GenBank OQ923251, Addgene 201804) was derived from pENTR4-TfR-sfGFP-myc tag-SpyCatcher003^19^ (GenBank MN433890 and Addgene 133451) by Gibson assembly. pcDNA3.1 HA-MBP-SnoopLigase2 N775Q-KDEL (GenBank OQ923252, Addgene 201805) was created by Gibson assembly, with the organization signal sequence-HA tag-maltose binding protein-SnoopLigase2 (including the N775Q mutation to remove a potential N-linked glycosylation site)-KDEL (to retain protein in the endoplasmic reticulum), based on pcDNA3.1-SpyTag-RBD^55^ (GenBank MT945427, Addgene 159999). This vector used the Influenza A/Guangdong/2017 H7 signal sequence present in the vector. pcDNA3.1 RBD-DogTag2-SpyTag (GenBank OQ923253, Addgene 201806) was derived from pcDNA3.1-SpyTag-RBD (Wuhan variant) (GenBank MT945427, Addgene 159999) by Gibson assembly. pcDNA3.1 RBD-DogTag2 NA-SpyTag (the non-reactive N854A mutant) was derived from pcDNA3.1 RBD-DogTag2-SpyTag by Gibson assembly. pET28a SUMO-DogTag2 (GenBank OQ923254, Addgene 201807) was derived from pET28a SUMO-DogTag (GenBank MG867376, Addgene 105629)^10^ by Gibson assembly. pET28a-HaloTag7-SnoopLigase2 (GenBank OQ923249, Addgene 201808) was derived from pET28a-HaloTag7-SnoopLigase^10^ (GenBank MG867371, Addgene 105627) by Gibson assembly. pDEST14-SpyCatcher003 S49C (Addgene 133448) was previously described.^19^ pET28a-SnoopTagJr-sfGFP (Addgene 201809) and pET28a-SnoopTag2-sfGFP (GenBank OQ923256, Addgene 201810) were derived from pET28a-SpyTag003-sfGFP^19^ (Addgene 133454). pET28 Affi-SnoopCatcher was created by cloning an anti-HER2 affibody^32^ onto the N-terminus of pET28-SnoopCatcher and described previously.^14^ pDEST14-DogCatcher was described previously^15^ (GenBank MZ365292, Addgene 171772).

TG2 constructs were designed with a C-terminal His_10_-tag, preceded by a flexible Gly/Ser-rich linker. Residue numbers of TG2 variants are based on the numbering of TG2 from PDB 2Q3Z.^56^ A gBlock of human TG2 (codon-optimized for expression in *E. coli*) was inserted into the pET28a backbone to create pET28a-TG2. pET28a-TG2_465_ was generated by truncation to include aa 1–465 of pET28a-TG2. pET28a-TG2_465_ C277S (GenBank OQ923257) was generated by mutation of the catalytic Cys277 to Ser. pET28a-DogTag2-TG2_465_ and pET28a-TG2_465_-DogTag2 were generated by introduction of DogTag2 and a GSS linker to the N- or C-terminus of pET28a-TG2_465_ as annealed complementary oligonucleotides. Oligonucleotides were annealed by incubating 2 μM of each oligonucleotide in 30 mM HEPES pH 7.5 and 100 mM potassium acetate at 94 °C for 2 min, then cooling to 25 °C. pET28a-SnoopTag2-TG2_465_ and pET28a-TG2_465_-SnoopTag2 were generated by introduction of SnoopTag2 and a GSS linker to the N- or C-terminus of pET28a-TG2_465_ as primer leader sequences. pET28a-DogTag2-TG2x (TG2x is TG2_465_ C230S) (GenBank OQ923258), pET28a-TG2x-DogTag2 (GenBank OQ923259, Addgene 201811), pET28a-SnoopTag2-TG2x (GenBank OQ923260), and pET28a-TG2x-SnoopTag2 (GenBank OQ923261) were generated by mutation of Cys230 to Ser in the corresponding construct. pET28a-SnoopTag2-TGFα (GenBank OQ923262, Addgene 201812) was designed with human TGFα (codon-optimized for *E. coli*) with a C-terminal sequence consisting of GSSGSS, His_6_-tag, SSG, and C-tag. pET28a-TG2x-TGFα (GenBank OQ923263) was generated by replacement of SnoopTag2 with human TGFα (codon-optimized for *E. coli*) at the C-terminus of pET28a-TG2x-SnoopTag2. pGEX-2T-GST-BirA^57^ (glutathione-*S*-transferase linked to biotin ligase) was a gift from Chris O’Callaghan, University of Oxford. pET28a-MBP-sTEV (Addgene 171782)^19^ is a modified TEV protease construct for high stability and activity in the absence of reducing agent. Inserts of all constructs were verified by Sanger sequencing (Source Bioscience).

### Protein Expression and Purification

SnoopTag-AffiHER2 variants, AviTag-DogTag-MBP variants, AviTag-DogCatcher-MBP, DogCatcher, Affi-SnoopCatcher, SUMO-DogTag variants, SnoopTag-sfGFP variants, TG2 variants, and TGFα constructs were expressed in *E. coli* BL21(DE3) RIPL. SnoopLigase variants and Cys-SnoopCatcher N847D were expressed in *E. coli* T7 Express (DE3). SpyCatcher003 S49C was expressed in *E. coli* C41 (DE3). Starter cultures were inoculated with single colonies into 10 mL LB containing either 100 μg/mL ampicillin (DogCatcher) or 50 μg/mL kanamycin (SnoopTag-AffiHER2 variants, AviTag-DogTag-MBP variants, AviTag-DogCatcher-MBP, SUMO-DogTag2, SUMO-DogTag2 and SnoopTag-sfGFP variants, Cys-SnoopCatcher N847D and SnoopLigase variants, TG2 variants, and TGFα constructs) and grown for 16 h at 37 °C with shaking at 200 rpm to create starter cultures. Expression was carried out by 1/100 dilution of the saturated starter culture in 1 L LB (for expressions in T7 Express cells) or 1 L LB + 0.8% (w/v) glucose [for expressions in BL21(DE3) RIPL and C41 (DE3) cells] plus appropriate antibiotic and grown at 37 °C with shaking at 200 rpm in ultra-yield baffled flasks (Thomson Instrument Company) until *A*_600_ 0.5. We then induced with 0.42 mM isopropyl β-d-1-thiogalactopyranoside (IPTG) at 30 °C with shaking at 200 rpm for 4 h. For TG2 and TGFα constructs, induction with 0.42 mM IPTG was carried out for 20 h at 18 °C. Cells were harvested by centrifugation and subsequently lysed by sonication on ice in Ni-NTA buffer (50 mM Tris–HCl pH 8.0 containing 300 mM NaCl) and 10 mM imidazole with mixed protease inhibitors (cOmplete mini EDTA-free protease inhibitor cocktail, Roche) and 1 mM phenylmethylsulfonyl fluoride (PMSF). Cell debris was removed by centrifugation in a JA25.50 rotor (Beckman) at 30,000–35,000*g* for 30–40 min at 4 °C, and the lysate was then incubated with Ni-NTA resin (Qiagen). After addition of the resin/lysate slurry to a Poly-Prep gravity column, the resin was washed with 10 column volumes of Ni-NTA buffer containing 10 mM imidazole three times, followed by elution using Ni-NTA buffer containing 200 mM imidazole.^57^ Subsequently, proteins were dialyzed into PBS (137 mM NaCl, 2.7 mM KCl, 10 mM Na_2_HPO_4_, and 1.8 mM KH_2_PO_4_) pH 7.5 using 3.5 kDa molecular weight cut-off dialysis tubing (Spectrum Labs). SnoopLigase, SnoopLigase2, and HaloTag-SnoopLigase variants were dialyzed into 25 mM Tris base adjusted to pH 7.4 by addition of solid boric acid and supplemented with 20% (v/v) glycerol. TG2 variants were dialyzed into HEPES-buffered saline (25 mM HEPES, 150 mM NaCl, pH 7.4). Before dialysis, the SnoopLigase2 prep should be adjusted to <300 μM to decrease the amount of aggregation upon dialysis. After dialysis, the samples were centrifuged at 17,000*g* for 10 min at 4 °C to remove potential aggregates, and the supernatant was used. Protein concentrations were determined from *A*_280_ using the extinction coefficient from ExPASy ProtParam.^58^

Expression and purification of MBP-sTEV were carried out as described above, except that the purification did not include EDTA-free protease inhibitor cocktail tablets. GST-BirA was expressed in *E. coli* BL21 (DE3) RIPL as above and purified using glutathione sepharose.^57^

Proteins were stored in aliquots at −80 °C. Typical protein yields per liter of culture are affibody fusions 4–10 mg, SnoopLigase2 8 mg, MBP fusions 20–25 mg, sfGFP fusions 15–35 mg, TG2 variants 1–3 mg, and SUMO fusions 20–25 mg.

### Insoluble Protein Expression and Refolding

TG2x-TGFα and SnoopTag2-TGFα were expressed in *E. coli* BL21(DE3). Starter cultures were inoculated from single colonies into 10 mL of LB containing 50 μg/mL kanamycin and grown for 16 h at 37 °C with shaking at 200 rpm. The saturated starter culture was diluted 1/100 in 1 L of LB plus 50 μg/mL kanamycin and grown at 37 °C with shaking at 200 rpm in ultra-yield baffled flasks (Thomson Instrument Company) to A_600_ 0.5. Expression was induced with 0.42 mM IPTG at 37 °C with shaking at 200 rpm for 4 h. Cells were harvested by centrifugation and subsequently lysed by sonication on ice in lysis buffer: 50 mM Tris–HCl pH 8.0, 300 mM NaCl, cOmplete mini EDTA-free protease inhibitor cocktail (Roche), and 1 mM PMSF. Inclusion bodies were pelleted by centrifugation in a JA25.50 rotor (Beckman) at 20,000*g* for 30 min at 4 °C to give the cleared lysate. The pellet was washed by resuspending in lysis buffer and repeating the previous centrifugation step. The pellet was solubilized by resuspending in denaturing buffer (8 M urea, 100 mM NaH_2_PO_4_, 10 mM Tris–HCl, and 2 mM 2-mercaptoethanol pH 8.0) and incubating for 16 h at 4 °C with gentle shaking. Insoluble debris was removed by centrifugation at 20,000*g* for 30 min at 4 °C to produce the solubilized pellet sample. Denatured recombinant protein was purified by Ni-NTA chromatography. 250 μL of packed volume Ni-NTA resin (Qiagen) was added directly to the samples as slurry and incubated for 1 h at 4 °C with gentle inversion. The resin was then washed with 30 column volumes of wash buffer (8 M urea, 100 mM NaH_2_PO_4_, and 10 mM Tris–HCl, pH 6.3), followed by 4 column volumes of elution buffer (8 M urea, 100 mM NaH_2_PO_4_, and 10 mM Tris–HCl, pH 4.5).

Purified protein was refolded by the rapid dilution method. Protein samples were added rapidly to ice-cold refolding buffer (100 mM NaH_2_PO_4_ adjusted to pH 9.7, 1.5 mM reduced glutathione, 0.3 mM oxidized glutathione, 1 mM PMSF, and 5 mM EDTA) at a ratio of 1:4.3 to give a final concentration of 1.5 M urea. The samples were incubated at 4 °C for 24–36 h without agitation, before concentration in a Vivaspin 20 molecular weight cut-off (MWCO) 5 kDa. Refolded protein was purified from the sample by size exclusion chromatography on a HiLoad 16/600 Superdex 200 pg column pre-equilibrated with HEPES-buffered saline (HBS: 10 mM HEPES and 150 mM NaCl, pH 7.4) running buffer, selecting the monomer peak (Figure S11B). The typical yield of SnoopTag2-TGFα was 1 mg per liter of culture.

### Biotinylation of Bait Proteins

Biotinylation of AviTag-containing proteins with GST-BirA was performed as described: a master mix was made of 100 μM target protein in 952 μL of PBS, 5 μL of 1 M MgCl_2_, 20 μL of 100 mM ATP, 20 μL of 50 μM GST-BirA, and a final concentration of 1.5 mM biotin.^57^ The reaction was incubated for 1 h at 30 °C with shaking at 800 rpm. An additional 20 μL of 50 μM GST-BirA was added, followed by a further 1 h incubation. Finally, the sample was dialyzed thrice in PBS pH 7.5 at 4 °C.

Biotinylation of Cys-SnoopCatcher N847D was carried out through modification with biotin-C2-maleimide (Anaspec). Cys-SnoopCatcher N847D was dialyzed into TBS (25 mM Tris + 150 mM NaCl) pH 7.2 + 1 mM tris(2-carboxylethyl)phosphine (TCEP) to maintain the protein in the reduced state. Cys-SnoopCatcher N847D was diluted to 100 μM in fresh TBS pH 7.2 + 2 mM TCEP and incubated at 25 °C for 30 min. Biotin-C2-maleimide was dissolved in anhydrous dimethyl sulfoxide (DMSO) to a final concentration of 100 mM. Biotin-C2-maleimide was added to a 10-fold molar excess over Cys-SnoopCatcher N847D and reacted at 25 °C with rotation for 4 h. Unreacted biotin maleimide was quenched by reaction with 1 mM dithiothreitol (DTT) for 30 min at 25 °C. Finally, the sample was dialyzed thrice in PBS pH 7.5 at 4 °C. We established that biotinylation was complete by a streptavidin gel shift assay.^57^

### Generation of SnoopTagJr and DogTag Libraries by Primer-Directed Site Saturation Mutagenesis

Site saturation mutagenesis was carried out using PCR with phagemids pBAD-DsbA(ss)-HA tag-SnoopTagJr KA-pIII for SnoopTagJr-based libraries and pBAD-DsbA(ss)-HA tag-DogTag NA-pIII for DogTag-based libraries. This procedure avoided any carryover of the reactive tag into the libraries. The libraries were assembled from two PCR fragments. In the first PCR, the forward primer was the mutagenic primer that introduced the mutations and replaced the reactive residue, while the reverse primer started from the ampicillin resistance gene (5′-GATCGTTGTCAGAAGTAAGTTGGCC-3′). In the second PCR, the forward primer hybridizes at the ampicillin resistance gene (5′-GGCCAACTTACTTCTGACAACGATC-3′), and the reverse primer extends from the plasmid immediately 5′ to the first residue being mutated. Two SnoopTagJr libraries were created: (i) library “S” with hard randomization at the underlined residues (734–737, 741, and 744–745) within SnoopTagJr—734-KLGSIEFIKVNK-745 with the forward primer 5′-CGACCTCGAGATCAGGGCNNKNNKNNKNNKATCGAATTCNNKAAAGTGNNKNNKGGATCCAGTGGTAGCGAAAACC-3′ where N is any one of the four bases and K is G or T and (ii) library “K” with soft randomization at the underlined residues within SnoopTagJr—734-KLGSIEFIKVNK-745 with the forward primer 5′-CGACCTCGAGATCAGGGCNWWNWWRVKRVKATCGAATTCNTTAAAGTGRVKRVKGGATCCAGTGGTAGCGAAAACC-3′ where R is A or G, V is A or C or G, and W is A or T. In both cases, the reverse library primer was 5′-GCCCTGATCTCGAGGTCG-3′. Two DogTag libraries were created: (i) library “C” with hard randomization at the underlined residues within DogTag—838-DIPATYEFTDGKHYITNEPIPPK-860 with the forward primer 5′-GCGATATTCCGGCTACATACGAATTCNNKNNKNNKNNKNNK NNKATCACCAATGAACCGATACCGC-3′ where N is any base and K is G or T and the reverse primer 5′-GAATTCGTATGTAGCCGGAATATCGC-3′ using the reverse primer design described above and (ii) library “E” with hard randomization at the underlined residues within DogTag: 838-DIPATYEFTDGKHYITNEPIPPK-860 and introducing an additional N-terminal residue to the Tag with the forward primer 5′-ACATACGAATTCACCGATGGTAAACATTATATCACCAATNNKNNKNNKCCGCCGAAAGGATCCAGTG-3′ and the reverse primer 5′-ATTGGTGATATAATGTTTACCATCGGTGAATTCGTATGTKNNKNNKNNKNNKNNGCCCTGATCTCGAGGTCG-3′. DpnI reaction was performed at 37 °C for 1 h, before inactivating at 80 °C for 20 min. The pBAD-DsbA(ss)-HA SnoopTagJr-pIII and pBAD-DsbA(ss)-HA DogTag-pIII libraries were constructed by Gibson assembly using the NEBuilder HiFi DNA Assembly Master Mix (New England Biolabs) with 0.2 pmol of both the insert and the backbone, incubating for 3 h. The reactions were purified using the Wizard SV Gel and PCR clean-up system (Promega) and eluted in nuclease-free water. 250 ng of the library DNA per cell aliquot was transformed by electroporation into the TG1 phage display electrocompetent *E. coli* (Lucigen). For each of the libraries, eight aliquots of 25 μL cells each were transformed in 0.2 mm cuvettes with a MicroPulser (both Bio-Rad) using program EC2. Immediately after electroporation, the cells were recovered in 1 mL recovery medium (Lucigen) pre-warmed to 37 °C. Cells were pooled and incubated for 1 h at 37 °C with shaking at 200 rpm. The recovered cells were plated onto four bioassay dishes (245 mm × 245 mm, Nunc) with LB agar containing 0.8% (w/v) glucose and 100 μg/mL carbenicillin and incubated for 16 h at 30 °C. The library cells were extracted from the plates by scraping and transferred to 2× TY containing 0.8% (w/v) glucose and 100 μg/mL carbenicillin, centrifuged at 3500*g* for 10 min, and stored in 2× TY containing 20% (v/v) glycerol at −80 °C.

### Phage Production and Purification

Each of the libraries in TG-1 cells was converted to phage-displayed protein libraries by infection with the M13KO7 helper phage (New England Biolabs). Varying volumes of 2× TY + 2% (w/v) glucose + 0.2% (v/v) glycerol + 100 μg/mL carbenicillin were inoculated with sufficient cells from the library to sample the library 5- to 10-fold. Cells were grown at 37 °C with shaking at 200 rpm until *A*_600_ reached 0.5. The cells were then infected with the M13KO7 helper phage with a multiplicity of infection of 20 at 37 °C, with shaking at 70 rpm for 45 min. The cells were then centrifuged at 4 °C for 10 min at 3000*g*, the supernatant decanted, and the cells resuspended in the same volume of 2× TY, 0.2% (w/v) l-arabinose, 0.2% (v/v) glycerol, and 100 μg/mL carbenicillin. The cells were then incubated for 30 min at 18 °C with shaking at 200 rpm, before addition of 50 μg/mL kanamycin. The culture was incubated at 18 °C with shaking at 200 rpm for 16 h. The cells were removed from the overnight cultures by centrifugation at 4000*g* for 15 min at 4 °C. The phage was precipitated from the supernatant by incubation with 4% (w/v) poly(ethylene glycol) average molecular weight 8000 (PEG8000, Thermo Fisher) + 0.5 M NaCl on ice for at least 1 h. The phage pellet was collected by centrifugation at 15,000*g* and 4 °C for 45 min and resuspended in PBS pH 7.4, with centrifugation at 15,000*g* and 4 °C to remove the insoluble material. Phage precipitations were repeated twice, with purified phage stored in PBS pH 7.4 supplemented with 20% (v/v) glycerol at −80 °C. The phage titer for the purified phage libraries was determined by quantitative PCR (qPCR) with a 2× SensiMix (Bioline) master mix performed on a Mx3000P qPCR system (Agilent). Data were analyzed using MxPro qPCR software version 4.10 (Agilent). qPCR reactions were carried out using a forward primer 5′-ACTGATTACGGTGCTGCTATCG-3′ and reverse primer 5′-TATCACCGTCACCGACTTGAGC-3′ and quantitated relative to a dilution series of M13KO7 (New England Biolabs).

### Catcher-Based Phage Display Selection

Biotinylated SnoopCatcher N847D was used at the bait to react with the SnoopTagJr phage libraries. Biotinylated AviTag-DogCatcher-MBP was initially used as the bait for the DogTag phage libraries in this type of selection, but subsequently, most selections with DogTag-phage libraries were carried out using the ligase-based selection method, discussed below. In the first round, 10^12^ colony-forming units (cfu) of the phage were reacted with 200 nM biotinylated bait in phage reaction buffer [PBS pH 7.4 + 0.05% (v/v) TWEEN 20 supplemented with 3% (w/v) bovine serum albumin (BSA)] in 200 μL Protein LoBind tubes (Eppendorf) for 4 h at 25 °C. Then, we quenched by 30 min with 30 μM Affi-SnoopCatcher (SnoopTagJr libraries) or 30 μM DogCatcher (DogTag phage libraries) at 25 °C. The phage bound to biotinylated SnoopCatcher N847D were captured using 100 μL Dynabeads Biotin Binder (Thermo Fisher) magnetic beads that had been washed four times with phage reaction buffer per 200 μL reaction in a 96-well low-bind microtiter plate (Greiner cat no. 655161) that had been pre-blocked for 2 h at 25 °C with phage reaction buffer. The beads were split between four wells and washed four times with 200 μL/well phage reaction buffer, with the beads being captured using a 96-well microtiter plate magnetic separation rack (New England Biolabs). The beads were resuspended in 150 μL of phage reaction buffer/well to which 50 μL of the reaction was added. The biotinylated bait was captured on the beads by incubation in the plate at 4 °C for 1 h with shaking at 700 rpm in an Eppendorf ThermoMixer. Weakly bound phages were removed by washing the beads once with phage reaction buffer, followed by a wash with 200 μL of 0.2 M glycine-HCl pH 2.2, then four times with 150 μL 50 mM Tris–HCl pH 7.5 + 150 mM NaCl + 0.5% (v/v) TWEEN 20, and twice with PBS + 0.1% (w/v) BSA. Phages were eluted from beads by digestion with 50 μL of 72 μM MBP-sTEV in PBS pH 7.4 per 25 μL beads in a Protein LoBind tube at 34 °C for 2 h at 1000 rpm. Phages were rescued by infection of the eluted phage into 2 mL TG-1 cells at the mid-log phase (*A*_600_ = 0.5). Reproduction of fresh phage was carried out as described in “Phage Production and Purification”. The subsequent rounds were carried out in phage reaction buffer in a similar manner with the following modifications. In the second round, 2 × 10^11^ cfu phages were reacted with 100 nM biotinylated bait for 30 min at 25 °C, followed by 30 min of quenching with 30 μM non-biotinylated bait at 25 °C. In the third round, 10^11^ cfu phages were reacted with 50 nM biotinylated bait for 10 min at 25 °C, followed by 30 min of quenching with 30 μM non-biotinylated bait at 25 °C. In the fourth round, phage reaction buffer was supplemented with 25% (v/v) *E. coli* BL21 cell lysate to disfavor non-specific binders, with 2 × 10^10^ cfu phage reacted with 10 nM bait and 5 min reaction, followed by 30 min of quenching with 30 μM non-biotinylated bait, all at 25 °C. The bacterial cell lysate was generated from untransformed *E. coli* BL21 (DE3) RIPL that had been grown in LB medium to *A*_600_ 0.5 and incubated with 0.42 mM IPTG at 18 °C and 200 rpm for 16 h. We added 2 mL of 50 mM Tris–HCl pH 7.5 + 300 mM NaCl (per gram of wet cell weight) supplemented with cOmplete Mini EDTA-free Protease Inhibitor Cocktail and 1 mM PMSF, and cells were lysed by sonication on ice at 50% duty cycle for 4 × 1 min, with 1 min rest after each run. After clarification of the lysate by centrifugation at 30,000*g* for 30 min at 4 °C, we adjusted to pH 7.5 with 1 M Tris–HCl pH 8.0, and the sample was stored at −80 °C prior to use. In the fifth round, the phage reaction buffer was supplemented with 25% (v/v) *E. coli* BL21 cell lysate, with 2 × 10^10^ cfu phage reacted with 1 nM bait. Reaction proceeded for 5 min, followed by 30 min quench with 30 μM non-biotinylated bait all at 25 °C. After each round, clones were picked, and plasmids were sequenced to determine the tags that had been selected.

### Ligase-Based Phage Display Selection

Here, the selection is for reaction of the tag displayed on the phage with the other tag on a biotinylated soluble protein in the presence of non-biotinylated SnoopLigase. Biotinylated AviTag-SnoopTagJr-AffiHER2 was used as bait with DogTag phage libraries. The SnoopLigase reaction buffer was 50 mM Tris-borate pH 7.4 + 3% (w/v) BSA + 0.05% (v/v) TWEEN 20. In the first round, 1 × 10^12^ cfu phage was reacted with 1 μM biotinylated bait and 20 μM SnoopLigase at 25 °C in 200 μL in Protein LoBind tubes (Eppendorf) for 24 h. Reactions were then precipitated by incubation with 4% (w/v) poly(ethylene glycol) average molecular weight 8000 (PEG8000, Thermo Fisher) + 0.5 M NaCl on ice for 1 h. The phage pellet was collected by centrifugation at 15,000*g* and 4 °C for 15 min and resuspended in phage reaction buffer. Phage bound to biotinylated bait were captured using 100 μL of BSA-blocked Dynabeads Biotin Binder (Thermo Fisher) per 200 μL reaction in a 96-well low bind Nunc plate that had been pre-blocked for 2 h at 25 °C with phage reaction buffer. The beads were split between four wells and washed four times with 200 μL/well phage reaction buffer, with the beads being captured using a 96-well microtiter plate magnetic separation rack (New England Biolabs) and finally resuspended in 150 μL phage reaction buffer/well to which 50 μL of the reaction was added. The biotinylated bait was captured on the beads by incubation in the plate at 4 °C for 1 h with shaking at 700 rpm in an Eppendorf ThermoMixer. Weakly bound phages were removed by washing the beads once with phage reaction buffer, followed by a wash with 200 μL of 0.2 M glycine-HCl pH 2.2, then four times with 150 μL of 50 mM Tris–HCl pH 7.5 + 150 mM NaCl with 0.5% (v/v) TWEEN 20, and twice with PBS + 0.1% (w/v) BSA. Phages were eluted from beads by digestion with 50 μL of 72 μM MBP-sTEV in PBS pH 7.4 per 25 μL beads in a Protein LoBind tube at 34 °C for 2 h at 1000 rpm. Phages were rescued by infection of the eluted phage into 2 mL of TG-1 cells at the mid-log phase (*A*_600_ = 0.5). Reproduction of fresh phage was carried out as described under “Phage Production and Purification”. The subsequent rounds were carried out in 50 mM Tris-borate buffer pH 7.4 + 3% (w/v) BSA + 0.05% (v/v) TWEEN 20 in a similar manner with the following modifications. In the second round, 1 × 10^11^ cfu phages were reacted with 0.5 μM biotinylated bait and 10 μM SnoopLigase for 4 h at 25 °C. In the third round, 2 × 10^10^ cfu phages reacted with 100 nM biotinylated bait and 5 μM SnoopLigase for 30 min at 25 °C. After each round, clones were picked, and plasmids were sequenced to determine the tags that had been selected.

### Fluorophore Conjugation to Cysteine-Containing SpyCatcher Proteins

SpyCatcher003 S49C was dialyzed into TBS pH 7.2 + 1 mM TCEP to maintain the protein in the reduced state. SpyCatcher003 S49C was diluted to a final concentration of 100 μM in fresh TBS pH 7.2 + 2 mM TCEP and incubated at 25 °C for 30 min. DyLight 680-maleimide (Thermo Fisher) or Alexa Fluor 647-maleimide (Thermo Fisher) was dissolved in anhydrous DMSO to a final concentration of 10 mg/mL, and samples were aliquoted and stored at −80 °C until use. Dye maleimide constructs were added to the protein at a 3-fold molar excess, with samples rapidly pipetted to mix thoroughly, followed by rotation end-over-end at 25 °C for 4 h, with tubes wrapped in foil to minimize light exposure. The excess unreacted dye was quenched by addition of 1 mM DTT and incubated at 25 °C for 1 h. Samples were centrifuged at 16,000*g* for 5 min at 4 °C to remove any aggregates. A volume of pre-swollen Sephadex G-25 resin (Sigma-Aldrich) 5-fold greater than the volume of the labeling reaction was added to a Bio-Rad Poly-Prep column and washed with 4 mL of PBS pH 7.4 to remove residual storage ethanol. After the PBS pH 7.4 drained from the column, dye-labeled samples were added to the top of the column to remove unconjugated dye. 1 mL of PBS pH 7.4 was added to the top of the column, and 300 μL fractions were collected. Fractions 1 and 2 were pooled and dialyzed thrice for at least 3 h in PBS pH 7.4 at 4 °C.

### Cell Line Transfection

Three component expressions were carried out with either (i) TfR-sfGFP-SnoopTag2 plus HA-MBP-SnoopLigase2 N775Q-KDEL plus RBD-DogTag2-SpyTag or (ii) TfR-sfGFP-SnoopTag2 plus HA-MBP-SnoopLigase2 N775Q-KDEL plus RBD-DogTag2 NA-SpyTag in Expi293F cells (Thermo Fisher) cultured in Expi293 expression media (Thermo Fisher). Cells at a density of 3.0 × 10^6^ cells/mL that had previously been growing in a medium supplemented with 50 U/mL penicillin/streptomycin (Thermo Fisher) were transfected in Expi293 expression media with no antibiotics present. Plasmids were transiently transfected with 2.7 μL of ExpiFectamine 293 Reagent per 1 μg of plasmid DNA with equal amounts of each plasmid added in each of the three component transfections. A mock transfection where the plasmid DNA was omitted was also carried out in parallel. Cells were grown in a humidified Multitron Cell incubator (Infors HT) at 37 °C with 7% (v/v) CO_2_, rotating at 110–125 rpm. ExpiFectamine transfection enhancers (Thermo Fisher) were added 16–22 h after transfection. Cells were grown for 4 days and then analyzed.

### Flow Cytometry

Cells were washed thrice in FACS buffer [PBS pH 7.5, 1 mM EDTA, and 1% (w/v) BSA] with centrifugation at 300*g* at 4 °C for 5 min. 10^6^ cells were incubated with a 500 nM SpyCatcher003-Alexa Fluor 647 for 60 min on ice in FACS buffer, followed by washing thrice in FACS buffer. Cells were maintained at 4 °C before analysis. Cells were analyzed on a BD Fortessa X20, gating on live cells based on forward scatter, side scatter, and 4′,6-diamidino-2-phenylindole (DAPI, Life Technologies) staining. DAPI was added after the above washes were carried out. Settings were a 405 nm laser and a 450/50 nm emission filter for DAPI, a 488 nm laser and a 530/30 nm emission filter for sfGFP, and a 640 nm laser and a 670/30 nm emission filter for Alexa Fluor 647. Data were analyzed using FlowJo version 9.0.

### Western Blot

3 × 10^6^ transfected Expi293F cells (sampled 5 days after transfection) were pelleted and resuspended in 1 mL of lysis buffer [20 mM Tris–HCl pH 7.4, 150 mM NaCl, 1% (v/v) Triton-X-100, 0.1% (w/v) SDS, 5 mM NaF, supplemented with cOmplete mini EDTA-free protease inhibitor cocktail, and 1 mM PMSF]. Cells were incubated on ice for 20 min, before centrifuging at 12,000*g* at 4 °C for 20 min. The supernatant was mixed with 6× SDS loading buffer containing 12 mM DTT and heated for 6 min at 95 °C, before resolving on 16% (w/v) SDS–PAGE using the XCell SureLock system (Thermo Fisher) at 180 V. Samples were transferred to the nitrocellulose membrane using the iBlot2 Dry Blotting System (Thermo Fisher) according to the manufacturer’s instructions at 25 V for 10 min. The membrane was blocked in PBS pH 7.4 with 0.05% (v/v) TWEEN 20 (PBST) with 5% (w/v) skimmed milk for 1 h before reaction with 60 nM SpyCatcher003-DyLight680 in PBST with 5% (w/v) skimmed milk for 2 h at 25 °C, with the membrane protected from light. The membrane was washed 4 × 5 min in PBST, followed by a 5 min wash in PBS, all the time protecting the membrane from light. Blots were imaged using a LI-COR Odyssey Fc, and image analysis was conducted using Image Studio Lite 5.2 (LI-COR). Pre-stained molecular weight markers (Thermo Scientific) were visible in the same channel as DyLight680.

### Conjugation of TG2 to Cargo

10 μM TG2x-DogTag2 was incubated with 20 μM SnoopLigase2 and 30 μM cargo protein at 2 mL in HBS at 4 °C for 16 h with gentle rotation. To confirm completion of the reaction, samples were analyzed by SDS–PAGE with Coomassie staining. % coupling to TG2x was calculated based on the depletion of the band of tagged TG2x: 100 × [1 – (tagged TG2x with SnoopLigase2)/(tagged TG2x without SnoopLigase2)]. The conjugated product was separated from unreacted cargo by size exclusion chromatography. 2 mL of samples was applied to a previously equilibrated Superdex 200 HiLoad 16/600 column (Cytiva), controlled by an ÄKTA pure (Cytiva) at 4 °C, with HBS as the running buffer. The peak corresponding to the conjugate product was concentrated in a Vivaspin 20 MWCO 30 kDa and analyzed by SDS–PAGE with Coomassie staining.

### Tag Comparison Assays

25 μM equimolar reactions of SnoopLigase2 with tagged proteins were performed in Tris-borate buffer (90 mM Tris base + 90 mM boric acid) pH 7.4 at 21 °C for 3 h in a total volume of 20 μL. Reactions were quenched by addition of 6× SDS loading buffer and 100 mM DTT. Samples were heated at 95 °C for 3 min and then run on a 16% (w/v) SDS–PAGE with Coomassie staining.

### Set Comparison Assays

25 μM equimolar reactions of SnoopLigase or SnoopLigase2 and tagged proteins were performed in PBS pH 7.4 or Tris-borate pH 7.4 + 15% (v/v) glycerol at 21 °C in a total volume of 100 μL. 9 μL samples were taken at desired time points and quenched with 6× SDS loading buffer. 100 mM DTT was added, and samples were heated at 95 °C for 3 min and then run on 16% SDS–PAGE before Coomassie staining.

Coupling efficiency (%) was calculated from band intensities as 100× product/(product + unreacted DogTag variant + unreacted SnoopTag variant) for three replicates. Old and new sets were compared by plotting product formation over time for each replicate to predict the time taken to reach 10% product in Excel. Mean and standard deviation were calculated from the three replicates.

### Analyzing the Effect of Reaction Conditions on SnoopLigase2 Reactivity

Proteins were incubated at 20 μM of each component in 10 mM HEPES pH 7.5 at 25 °C, unless indicated otherwise. To measure pH dependence, reactions were performed at 25 °C for 9 min in succinate–phosphate–glycine (12.5 mM succinic acid, 50 mM sodium dihydrogen phosphate, and 44 mM glycine) to allow good buffering over a wide range of pH values. To measure temperature dependence, reactions were performed for 10 min. To measure dependence on NaCl, reactions were performed for 10 min with the indicated additional concentration of NaCl added to the original buffer. To measure additive dependence, reactions were performed in PBS pH 7.4 for 8 min with 1% (v/v) Triton X-100, 1% (v/v) TWEEN 20, 1 mM EDTA, or 2 mM DTT. To measure buffer dependence, reactions were performed for 12 min with 90 mM Tris-borate, 100 mM sodium phosphate buffer (8:2 ratio of Na_2_HPO_4_ to NaH_2_PO_4_), 10 mM HEPES, or PBS, all at pH 7.5. Buffers were added to the reactions as 10× stock solutions. To terminate the reaction, we added 6× SDS loading buffer [0.23 M Tris–HCl, pH 6.8, 24% (v/v) glycerol, 120 μM bromophenol blue, and 0.23 M SDS]. DTT was added to 100 mM, and then samples were heated in a thermocycler for 3 min at 95 °C. SDS–PAGE was performed at 190 V in 25 mM Tris–HCl, 192 mM glycine, and 0.1% (w/v) SDS. Gels were stained with InstantBlue Coomassie stain, destained with Milli-Q water, and imaged using a ChemiDoc XRS imager with ImageLab version 6.1.0 software (Bio-Rad). Product formation was quantified using ImageLab version 6.1.0.

### Biotin Cadaverine Assay for Transglutaminase Activity

Wells of a 96-well Nunc Maxisorp plate were coated with 200 μL of 100 μg/mL dimethyl casein (C9801, Sigma-Aldrich) in HBS for 16 h at 4 °C.^35^ Wells were then blocked with 300 μL Pierce protein-free TBS blocking buffer (37570, Thermo Scientific) for 1 h at 25 °C. Wells were washed thrice with 300 μL of HBS + 0.5% (v/v) TWEEN 20. Wells were then incubated with 200 μL of TG2 variant at the indicated concentration in HBS, 1 mM DTT, 1 mM EZ-Link Pentylamine-Biotin (21345, Thermo Scientific), and 1 mM CaCl_2_ for 30 min at 37 °C. Wells were washed thrice with 300 μL of HBS + 0.5% (v/v) TWEEN 20 before incubation with 150 μL of 0.3 μg/mL Pierce high-sensitivity streptavidin-horseradish peroxidase (HRP) (21130, Thermo Scientific) diluted in HBS for 1 h at 25 °C. Wells were washed six times in HBS + 0.5% (v/v) TWEEN 20, before signal generation by adding 100 μL of 1-Step Ultra TMB-ELISA Substrate Solution (34029, Thermo Scientific). The reaction was stopped by addition of 100 μL of 1 M HCl, and *A*_450_ was measured on a FLUOstar Omega plate reader (BMG Labtech).

### Purification of the SnoopLigase2 Reaction Product

TG2x-DogTag2, SnoopTag2-sfGFP, and HaloTag-SnoopLigase2 were incubated at 10 μM each in HBS pH 7.4 in a total volume of 200 μL for 16 h at 24 °C. To capture HaloTag-SnoopLigase2, 25 μL of washed and equilibrated Magne HaloTag resin (Promega) was added, followed by TWEEN 20 at a final concentration of 0.01% (v/v). Samples were incubated for 1 h at 24 °C on a rotor. The resin was collected using a magnetic rack. After washing the resin twice with 100 μL of HBS pH 7.4 + 0.01% (v/v) TWEEN 20, then thrice with 100 μL of HBS pH 7.4, the resin was resuspended in one of the three buffers for the elution step. For acid elution, the resin was resuspended in 25 μL of 50 mM glycine–HCl pH 2.5 and incubated in a ThermoMixer (Eppendorf) for 1 min at 37 °C with 800 rpm shaking. The resin was collected with a magnetic rack, and the eluent was removed and neutralized by addition of 2.5 μL 1 M Tris–HCl pH 9.5. Acid elution was then repeated twice more. For imidazole elution, the resin was resuspended in 25 μL of HBS with 3 M imidazole adjusted to pH 7.4. The resin was incubated in a ThermoMixer for 4 h at 37 °C with 800 rpm shaking. The resin was collected, and the eluent was removed. Elution was then repeated twice more. For peptide elution, the resin was resuspended in 25 μL of PBS pH 7.4, with the DogTag:SnoopTagJr conjugated product^10^ and incubated in a ThermoMixer for 4 h at 37 °C with 800 rpm shaking. The resin was collected and the eluent was removed. Elution was then repeated twice more. Beads were then resuspended in 50 μL of PBS pH 7.4 and 6× SDS loading buffer and heated at 98 °C for 3 min to release any proteins bound to the bead-coupled HaloTag-SnoopLigase2.

### Mass Spectrometry

40 μM SUMO-DogTag2 and 200 μM SnoopTag2 solid-phase synthesized peptide (GKLGYIEFYKVEKGY, Insight Biotechnology at 95% purity) were incubated with 60 μM SnoopLigase2 in TB pH 7.4 (50 mM Tris base adjusted to pH 7.4 with boric acid) and 15% (v/v) glycerol in a total volume of 200 μL for 24 h at 4 °C. The full 200 μL reaction was loaded onto a HiLoad 16/600 Superdex 200 pg column pre-equilibrated with HBS pH 7.4 running buffer. The relevant peak was collected and concentrated in a Vivaspin20 spin concentrator (Sartorius) with 5 kDa MWCO. Analysis of this reaction was performed using a RapidFire 365 platform (Agilent) comprising a jet-stream electrospray ionization source coupled to an Accurate-Mass Quadrupole Time-of-Flight (Q-TOF) (Agilent) detector. Data were analyzed using MassHunter Qualitative Analysis (Agilent). The expected mass of the SUMO-DogTag2:SnoopTag2 product was calculated by combining the mass of SUMO-DogTag2 (minus initiating formylmethionine) and SnoopTag2, as predicted by ExPASy ProtParam, and subtracting 17.0 Da to account for the loss of ammonia during isopeptide bond formation.

### Treatment and Washing of Cells

Confluent cells were washed twice with RPMI 1640 and serum-starved in RPMI 1640 + 1% (v/v) penicillin/streptomycin for 24 h prior to detachment with trypsin. Detached cells were washed once in RPMI 1640 + 10% (v/v) FBS to quench trypsin, then washed in RPMI 1640 + 1% (v/v) penicillin/streptomycin, and resuspended in RPMI 1640 + 1% (v/v) penicillin/streptomycin. 9 × 10^4^ DU145 cells were seeded and then incubated for 3 h to allow attachment. Once attached, protein in HBS pH 7.4 was added to a final concentration of 100 nM. Cells were incubated for 30 min, then selected samples were washed five times with RPMI 1640, and all samples were incubated for 3 days in RPMI 1640 + 1% (v/v) penicillin/streptomycin.

### Fluorescence Microscopy

9 × 10^4^ DU145 cells were seeded onto eight-well glass coverslips (80807, Ibidi), treated with protein, and washed as detailed above. After 3 days of incubation at 37 °C with 5% (v/v) CO_2_, cells were fixed for 20 min at 25 °C in PBS pH 7.4 + 4% (w/v) paraformaldehyde, permeabilized for 5 min at 25 °C in PBS pH 7.4 + 0.1% (v/v) Triton X-100, and then blocked for 30 min at 25 °C with PBS + 1% (w/v) BSA + 0.1% (v/v) TWEEN 20. For E-cadherin and vimentin staining, cells were stained with 1.25 μg/mL mouse anti-E-cadherin M168 (ab76055, Abcam) and rabbit anti-vimentin EPR3776 (ab92547, Abcam) for 1 h, followed by three washes with PBS-T (PBS pH 7.4 + 1% (v/v) TWEEN 20). Then, the cells were stained with 2.5 μg/mL goat anti-mouse Alexa Fluor 647 (A-21236, Thermo Fisher) and goat anti-rabbit Alexa Fluor 568 (A-11011, Thermo Fisher) secondary antibodies for 1 h, followed by three washes with PBS-T. All antibodies were diluted in PBS pH 7.4 + 1% (w/v) BSA + 0.1% (v/v) TWEEN 20. For phalloidin staining, cells were incubated with 5 U/mL Phalloidin CF647 (BT00041-T, Cambridge Bioscience) diluted in PBS pH 7.4 for 1 h, followed by three washes in PBS pH 7.4. All microscopy samples were counterstained with 1 μg/mL DAPI (Life Technologies) for 5 min prior to imaging.

Cells were imaged on an Olympus ScanR wide-field microscope with a scientific complementary metal-oxide-semiconductor (sCMOS) Hamamatsu Orca Fusion B camera using a 40× LCACHN air/dry numerical aperture (NA) 0.55 working distance (WD) 2.2 mm objective. Images were collected using ScanR Acquisition software (version 3.0.0). Excitation was performed with 395/20 excitation and 432/36 emission (DAPI), 575/25 excitation and 595/31 emission (Alexa Fluor 568), and 640/30 nm excitation and 698/70 nm emission (Alexa Fluor 647, CF647). Identical exposure times were used within a single channel for all samples. The typical exposure times were 50–200 ms. Images were cropped in ImageJ (version 1.54b) and show representative fields of view. All images in the same figure were prepared, collected, and analyzed using the same settings.

To quantify cell morphology, three images were taken per treatment condition of cells stained with Phalloidin CF647 (BT00041-T, Cambridge Bioscience) using 640/30 nm excitation and 698/70 emission. Visualization of actin filaments was used to manually segment the cells in ImageJ (version 1.54b) to calculate the aspect ratio, defined as (largest diameter along the major axis)/(largest diameter orthogonal to the major axis). Violin plots were generated from 51 cells per treatment condition using GraphPad Prism (version 9.5.0).

### Resazurin Assay

1.5 × 10^4^ DU145 cells were seeded into wells of a 96-well clear-bottom plate (165305, Thermo Fisher) and treated with protein and washes as detailed above. After 3 days of incubation at 37 °C with 5% (v/v) CO_2_, 10 μL of AlamarBlue HS cell viability reagent (A50100, Thermo Fisher) was added to 90 μL of cell culture, and cells were incubated for 60 min at 37 °C with 5% (v/v) CO_2_. Fluorescence was measured using a SpectraMax M3 (Molecular Devices) microplate reader with 560 nm excitation and 590 nm emission. The sample signal was blanked against a control sample of RPMI 1640 media + 10% (v/v) AlamarBlue HS cell viability reagent.

### Graphics and Sequence Analysis

Structures were visualized in PyMOL version 2.0.6 (DeLano Scientific) based on PDB 2WW8 for RrgA,^29^ PDB 7R6W for RBD,^59^ PDB 2Q3Z for TG2,^56^ and PDB 2B3P for sfGFP.^60^

### Statistics and Reproducibility

No statistical method was used to pre-determine sample sizes. No data were excluded from our analyses. Experiments were not randomized. The investigators were not blinded to allocation during the experiments and assessment of outcome. Statistical tests were performed in GraphPad Prism (version 9.5.0). Significance was analyzed by unpaired Student’s *t*-tests, except for Figure 7B, where unpaired Welch’s *t*-test was used because of unequal variance. *p < 0.05, **p < 0.01, and ***p < 0.001; ns, not significant.

## Data Availability

Amino acid sequences of SnoopLigase2, SnoopTag2, and DogTag2 are available in Figure S2. Sequences of these and other constructs are found in GenBank, as described in the section “Plasmids and Cloning”. Plasmids have been deposited in the Addgene repository (https://www.addgene.org/Mark_Howarth/), as described in the section “Plasmids and Cloning”. Further information and request for resources and reagents should be directed to and will be fulfilled by the lead contact, M.H.

## Supporting Information Available

Sequence alignments and additional characterization of protein samples and reactivity (PDF)
