# Supplementary material for: Design and Evolution of Enhanced Peptide–Peptide Ligation for Modular Transglutaminase Assembly
Source: Bioconjug Chem. 2023 Jun 8;34(6):1019–36. doi: 10.1021/acs.bioconjchem.3c00122 (PMC10288437; doi:10.1021/acs.bioconjchem.3c00122)
Supplement: Supplementary file 1 — bc3c00122_si_001.pdf [file bc3c00122_si_001.pdf]

Supporting Information

Design and evolution of enhanced peptide-peptide ligation for modular transglutaminase assembly

Anthony H. Keeble[†,‡,§], Dominic P. Wood[†,§], and Mark Howarth[†,‡,*]

[†]Department of Biochemistry, University of Oxford, South Parks Road, Oxford, OX1 3QU, UK.
[‡]Current address: Department of Pharmacology, University of Cambridge, Tennis Court Road, Cambridge, CB2 1PD, UK.
[§]These authors contributed equally.

*Corresponding author and Lead Contact:
Mark Howarth,
Department of Pharmacology,
University of Cambridge,
Tennis Court Road,
Cambridge,
CB2 1PD,
UK
E-mail: mh2186@cam.ac.uk

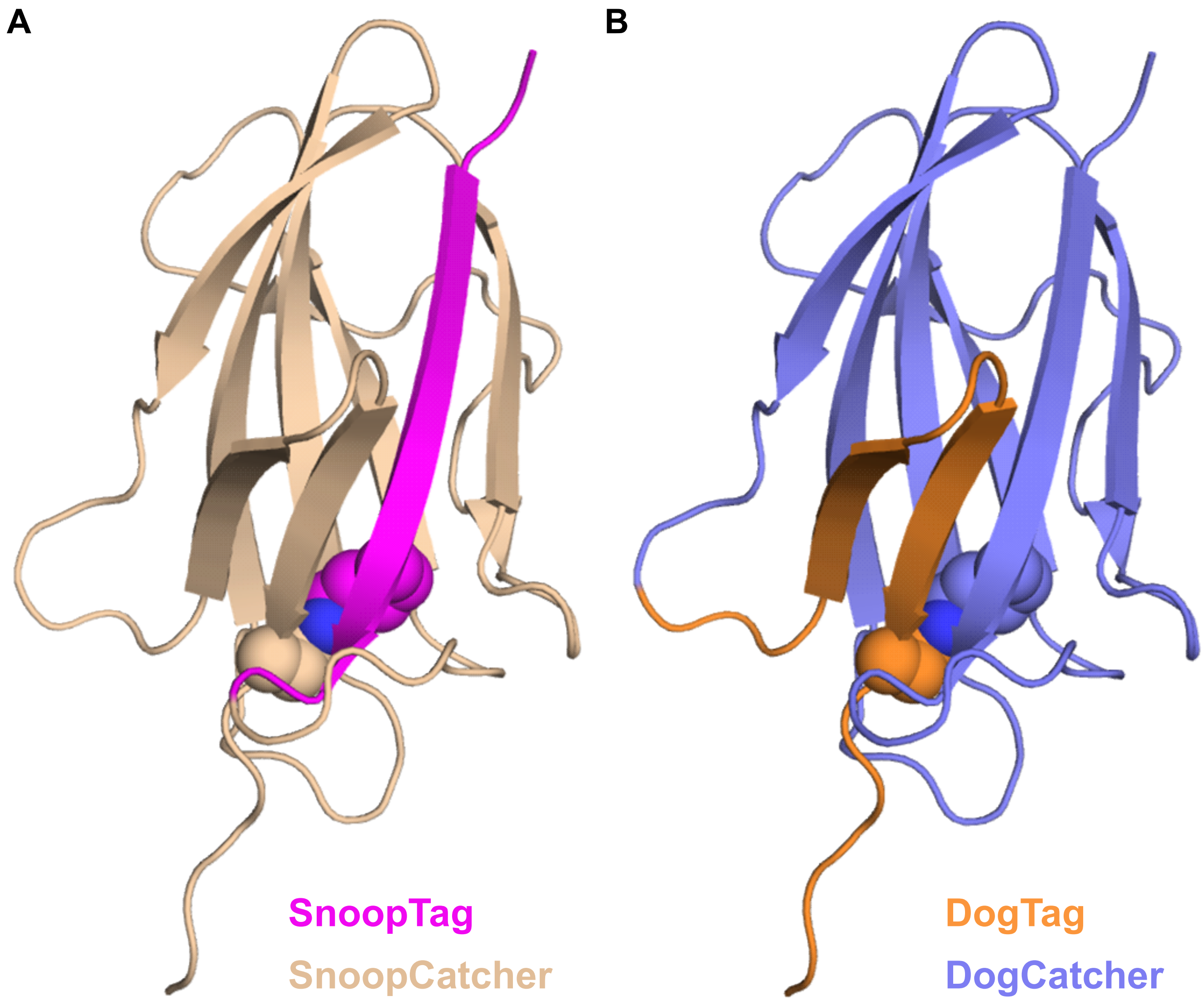

Figure S1. Structural comparison of the different Tag/Catcher pairs split from RrgA domain 4. (**A**) Schematic of SnoopTag/SnoopCatcher based on the structure of the parental domain from PDB 2WW8. The isopeptide-forming residues of SnoopTag (Lys742) and SnoopCatcher (Asn854) are shown in sphere format. The dark blue sphere indicates the nitrogen atom in the isopeptide bond. (**B**) Schematic of DogTag/DogCatcher as in (A).

A

```
              734  740        750        760        770        780
Domain 4      KLGDIEFIKVNKNDKKPLRGAVFSLQKQHPDYPDIYGAIDQNGTYQNVRTGEDGK
SnoopLigase            VNKNDKKPLRGAVFSLQKQHPDYPDIYGAIDQNGTYQNVRTGEDGK
SnoopLigase2           VNKNDKKPLRGAVFSLQKQHPDYPDIYGAIDQNGTYQNVRTGEDGK

               790        800        810        820        830        840
Domain 4      LTFKNLSDGKYRLFENSEPAGYKPVQNKPIVAFQIVNGEVRDVTSIVPQDIPAGY
SnoopLigase   LTFKNLSDGKYRLFENSEPPGYKPVQNKPIVAFQIVNGEVRDVTSIVPPGVPATY
SnoopLigase2  LTFKNLSDGKYRLIENSEPPGYKPVQNKPIVSFRIVDGEVRDVTSIVPPGVPATY

                    850        860
Domain4       EFTNDKHYITNEPIPPK-
SnoopLigase   EFT-
SnoopLigase2  EFT-
```

Reactive Lysine
Catalytic Glutamate
Reactive Asparagine
Mutations to make SnoopLigase
Mutations to make SnoopLigase2

B

```
              734  740
SnoopTag      KLGDIEFIKVNK
SnoopTagJr    KLGSIEFIKVNK
SnoopTag2     KLGYIEFYKVEK
```

Reactive Lysine
Mutation to create SnoopTagJr
Mutations to create SnoopTag2

C

```
                 840        850        860
Domain 4      DIPAGYEFTNDKHYITNEPIPPK
DogTag        DIPATYEFTDGKHYITNEPIPPK
DogTag2       DIPATYEFTDGKHYITNEPLPPK
```

Reactive Asparagine
Mutations to create DogTag
Mutation to create DogTag2

Figure S2. Amino acid sequence alignments of the new variants. (**A**) Alignment of RrgA domain 4 with SnoopLigase and SnoopLigase2. (**B**) Alignment of SnoopTag, SnoopTagJr and SnoopTag2. (**C**) Alignment of DogTag and DogTag2 with the same region in Domain 4. Numbering is based on PDB 2WW8.

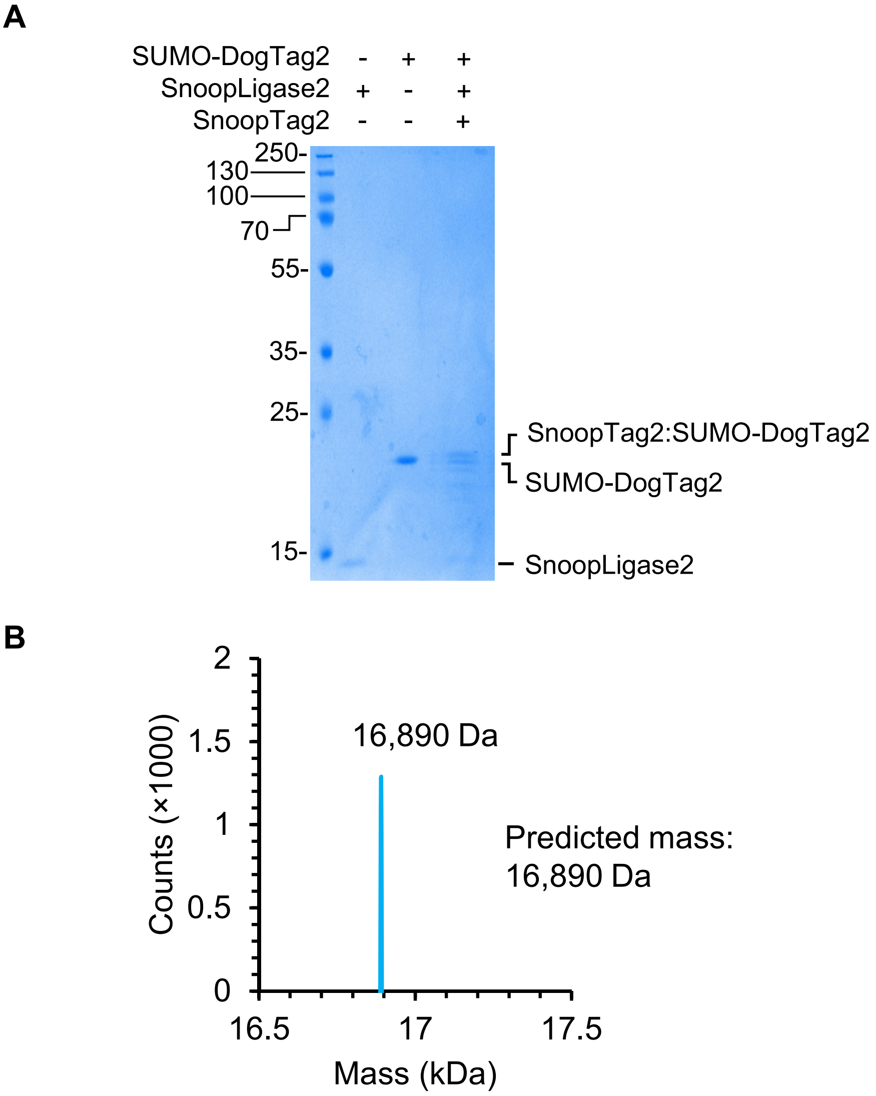

Figure S3. Mass spectrometry of the SnoopLigase2-mediated ligation. (**A**) Conjugation of SnoopTag2 to SUMO-DogTag2 by SnoopLigase2. 200 μM SnoopTag2, 40 μM SUMO-DogTag2 and 60 μM SnoopLigase2 were incubated for 24 h at 4 °C. Analysis by SDS-PAGE with Coomassie staining. (**B**) Electrospray ionization mass spectrometry detects a 16,890 Da product corresponding to SUMO-DogTag2:SnoopTag2 with loss of ammonia.

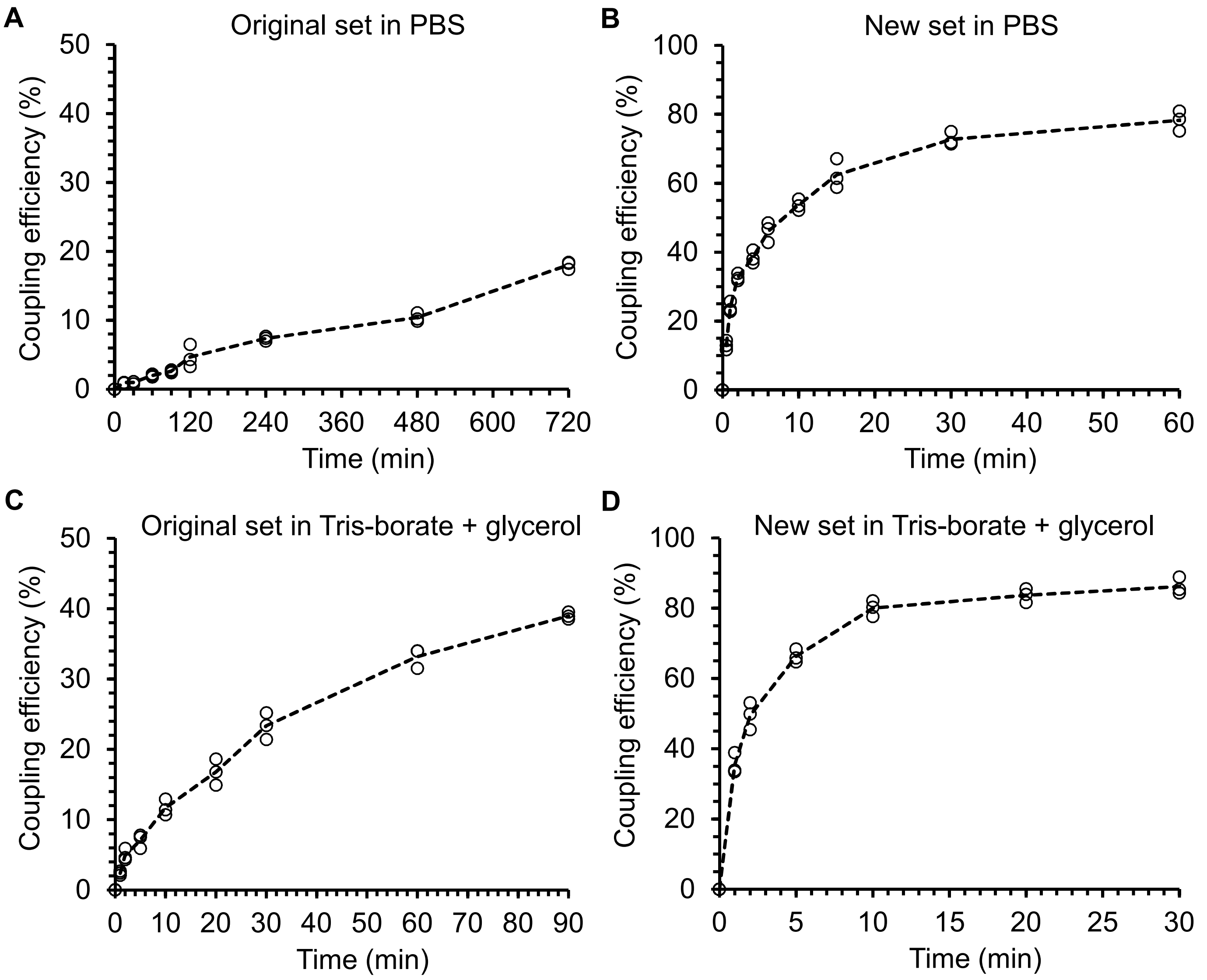

E

Components	Buffer	Time to 10% coupling (s)
SnoopLigase, SnoopTagJr, DogTag	PBS	27,120 ± 1,800
	Tris-borate + 15% (v/v) glycerol	510 ± 50
SnoopLigase2, SnoopTag2, DogTag2	PBS	22 ± 2
	Tris-borate + 15% (v/v) glycerol	15 ± 4

Figure S4. Reaction rate for original or second generation set. (**A**) Reaction of original set in PBS. SnoopTagJr-sfGFP, SUMO-DogTag and SnoopLigase (25 μM each) were incubated at 21 °C in PBS pH 7.4. Circles show each of the triplicate data-points. The dotted line connects each mean. (**B**) Reaction of new set (SnoopTag2-sfGFP, SUMO-DogTag2, SnoopLigase2) in PBS as in (A). (**C**) Reaction of original set in Tris-Borate with 15% (v/v) glycerol as in (A). (**D**) Reaction of new set in Tris-Borate pH 7.4 with 15% (v/v) glycerol as in (B). (**E**) Relative reaction rate based on the time to reach 10% coupling. Mean ± 1 standard deviation, n = 3.

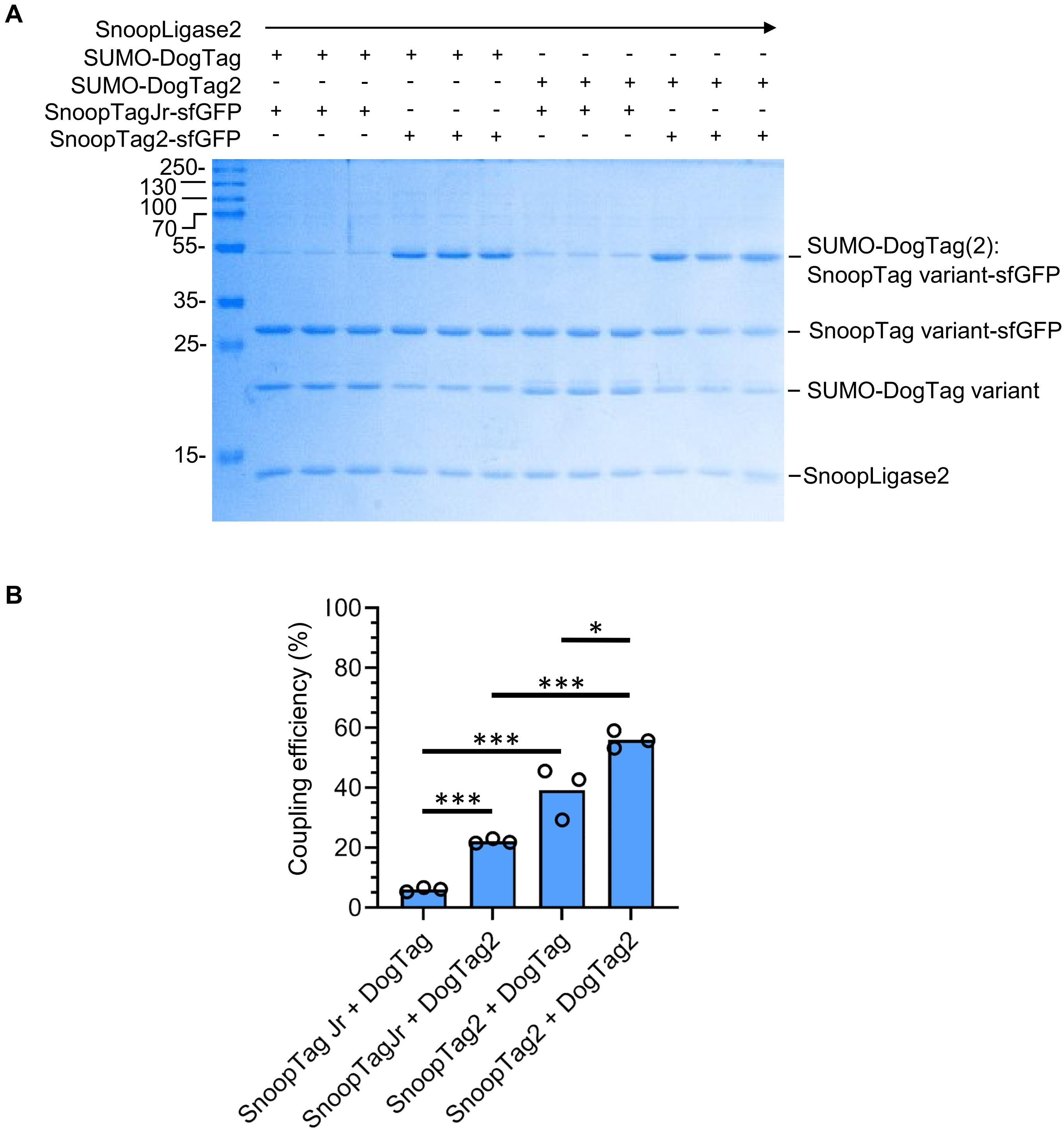

Figure S5. Reactivity of original versus modified tags for SnoopLigase2 reaction. (**A**) Reactions were performed in triplicate with each component at 25 µM in Tris-Borate pH 7.4 for 3 h at 21 °C, before SDS-PAGE/Coomassie. (**B**) Quantification of reaction in (A). Circles show each of the triplicate data-points for tagged proteins reacting with SnoopLigase2, with the bar indicating the mean. Unpaired two-tailed Student's t test was used to compare selected combinations of tags (* denotes $p < 0.05$, *** denotes $p < 0.001$, $n = 3$).

A

B

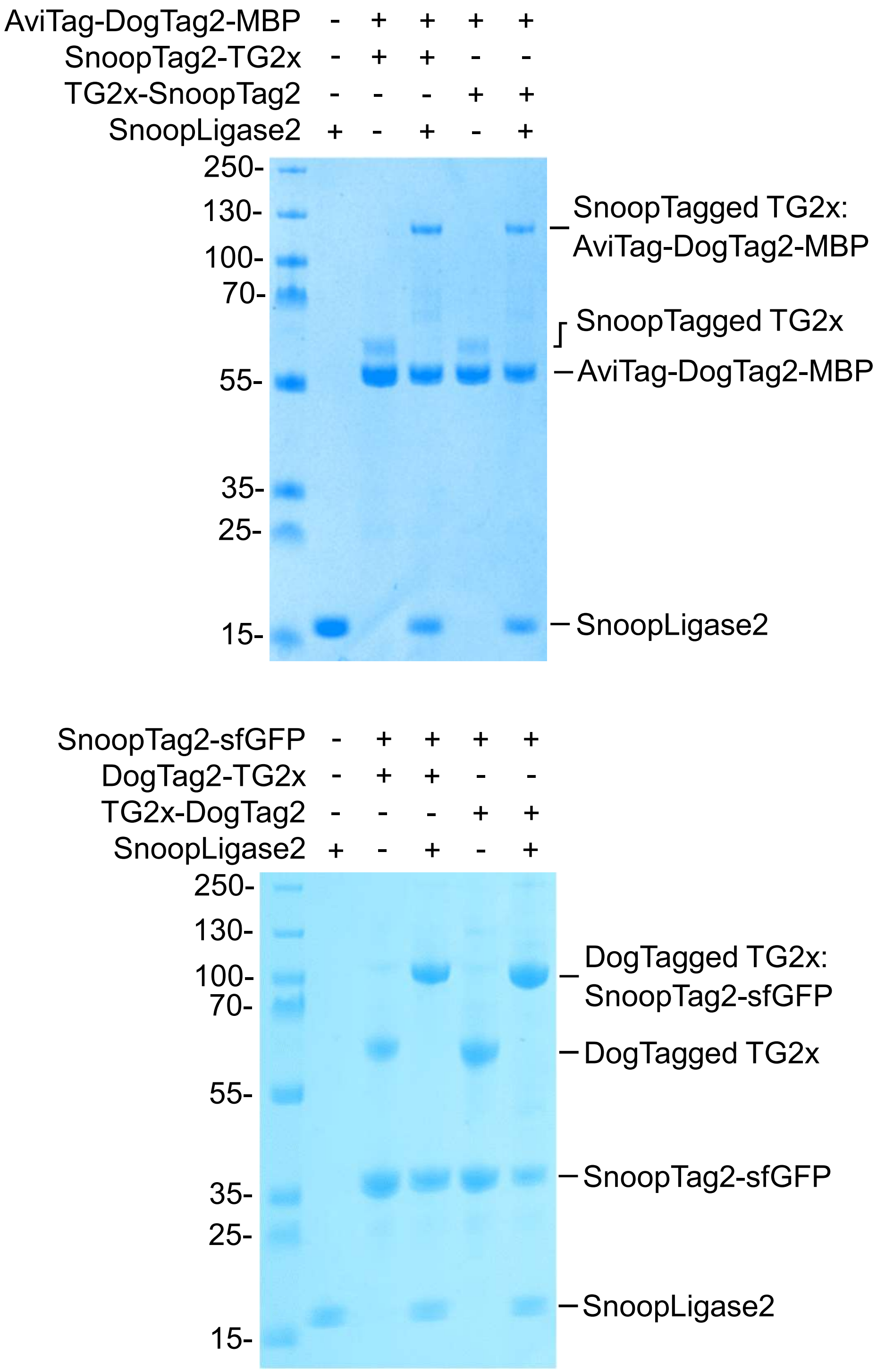

Figure S6. Efficient SnoopLigase2 ligation with the tags at either the N- or C-terminus. (**A**) SnoopTag2 can be ligated at the N- or C-terminus. 1 µM TG2x with SnoopTag2 at the N- or C-terminus was coupled with 7.5 µM AviTag-DogTag2-MBP by incubation with 5 µM SnoopLigase2 for 16 h at 4 °C in PBS pH 7.4. (**B**) DogTag2 can be ligated at the N- or C-terminus. 10 µM TG2x with DogTag2 at the N- or C-terminus was coupled with 30 µM SnoopTag2-sfGFP by incubation with 20 µM SnoopLigase2 for 16 h at 4 °C in PBS pH 7.4 (SDS-PAGE with Coomassie staining).

A

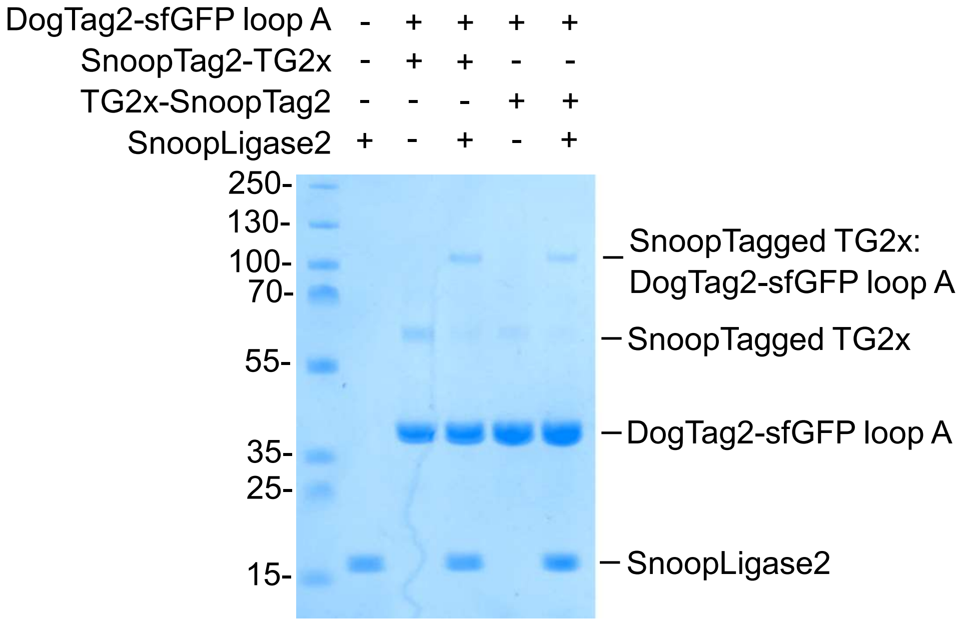

B

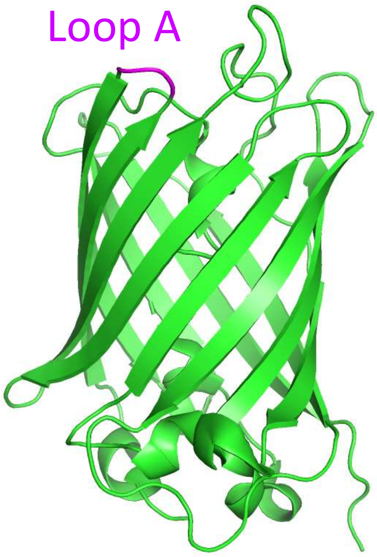

Figure S7. SnoopLigase2 ligation with tags at an internal site. (**A**) 2 μM TG2x bearing SnoopTag2 at the N- or C-terminus was coupled to 16 μM DogTag2-sfGFP loop A by incubation with 12 μM SnoopLigase2 for 16 h at 4 °C in PBS pH 7.4 (SDS-PAGE with Coomassie staining). (**B**) Structure of sfGFP (PDB 2B3P) with Loop A highlighted in magenta.

A

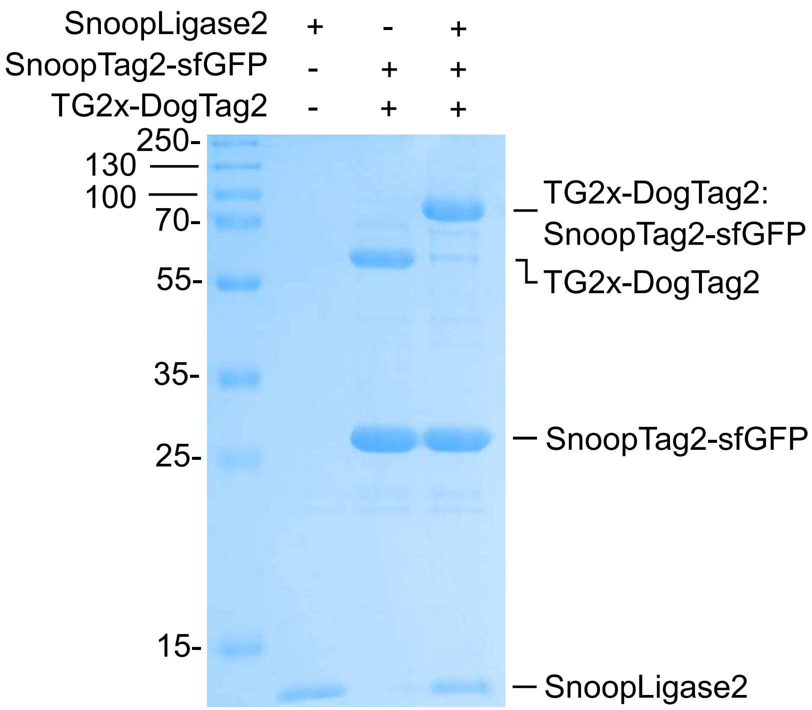

B

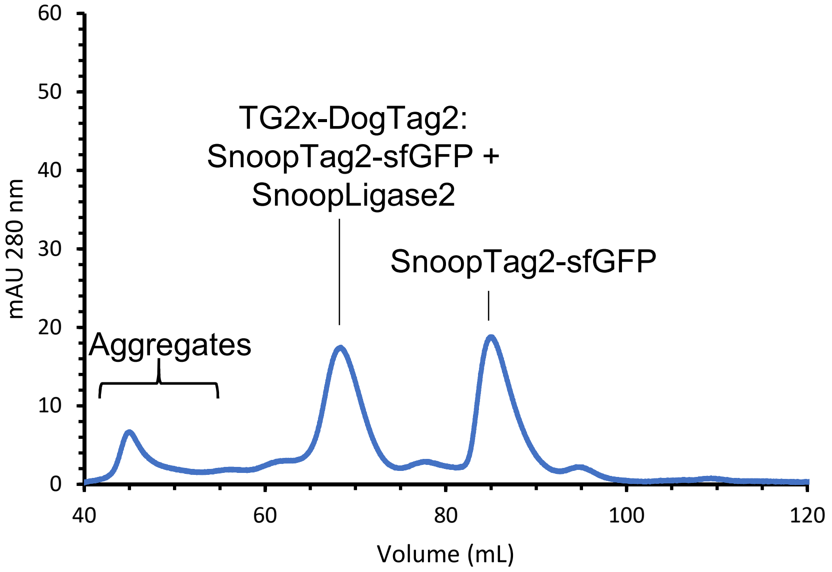

Figure S8. Preparation of TG2x:sfGFP ligation product. (**A**) SnoopLigase2 ligation reaction between TG2x-DogTag2 and SnoopTag2-sfGFP. 10 µM TG2x-DogTag2, 30 µM SnoopTag2-sfGFP and 20 µM SnoopLigase2 were incubated for 16 h at 4 °C in PBS pH 7.4. Reaction was analyzed by SDS-PAGE with Coomassie staining. (**B**) Size-exclusion chromatography trace of SnoopLigase2 ligation reaction. Reaction sample from (A) was loaded onto a Superdex 200 column. High molecular weight aggregates are commonly observed for both full length and truncated forms of TG2x. The product TG2x-DogTag2:SnoopTag2-sfGFP, non-covalently associated with SnoopLigase2, is separated from unreacted SnoopTag2-sfGFP substrate.

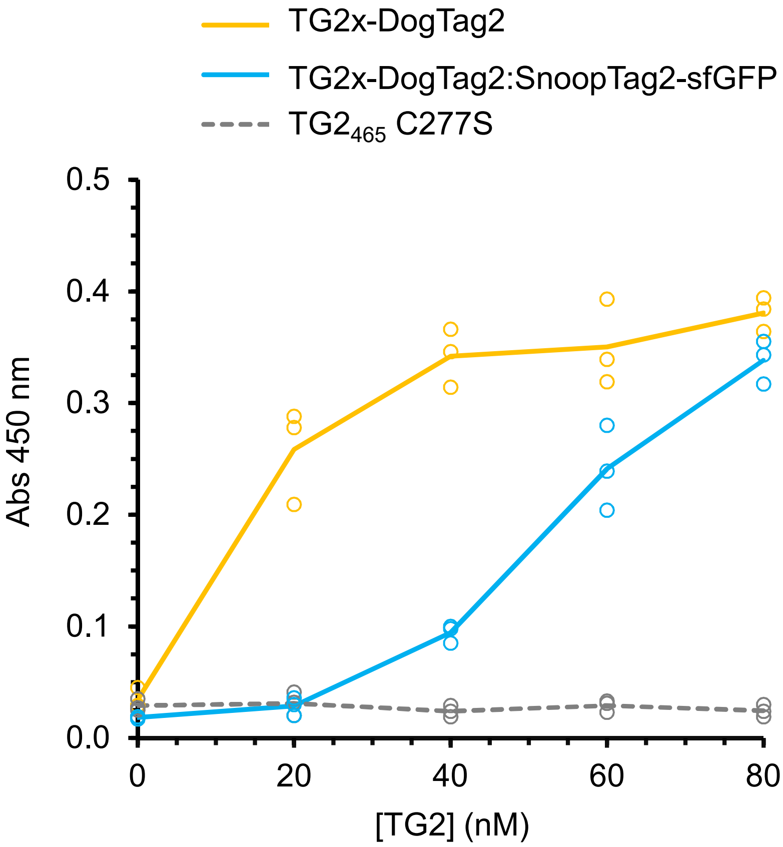

Figure S9. Transamidase activity of TG2:cargo conjugate. Cadaverine assay of the TG2x:cargo product from Fig. 6D after size-exclusion chromatography purification, compared to the original TG2x-DogTag2 or the $TG2_{465}$ C277S negative control. The assay was performed in HBS pH 7.4, 2 mM $CaCl_2$, 1 mM DTT and 1 mM biotin-cadaverine with the indicated concentration of TG2 variant for 30 min at 37 °C. Circles show each of the triplicate data-points, with the lines connecting the mean.

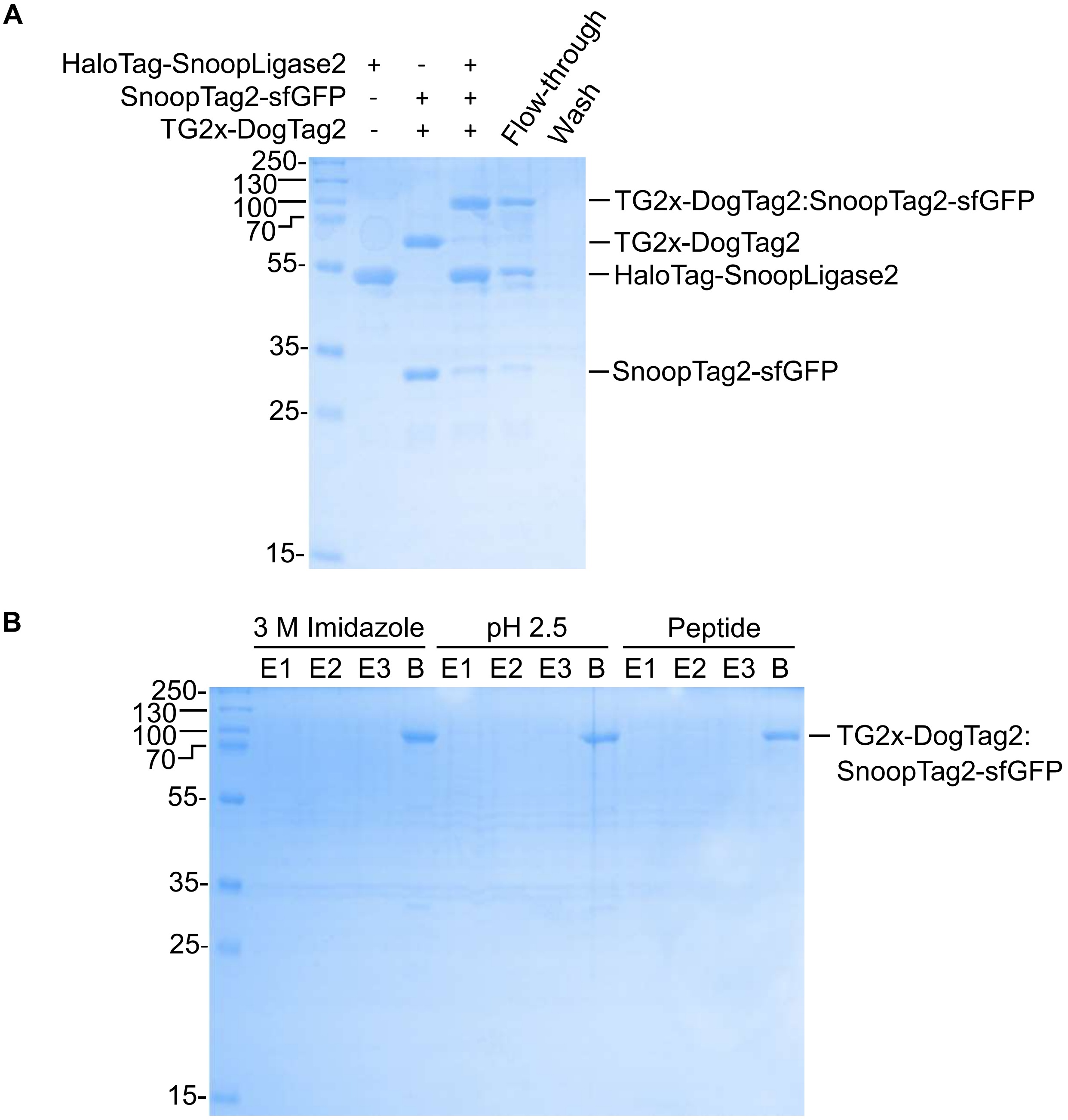

Figure S10. SnoopLigase2 is stably anchored to its reaction product. SnoopTag2- and DogTag2-linked proteins are covalently conjugated using HaloTag-SnoopLigase2. HaloLink-sepharose covalently captures HaloTag-SnoopLigase2 and then the complex is subjected to stringent wash buffers. (**A**) Conjugation of SnoopTag2- and DogTag2-linked proteins by HaloTag-SnoopLigase2 and capture of the complex by HaloLink beads. 10 μM each of TG2x-DogTag2, SnoopTag2-sfGFP, and HaloTag-SnoopLigase2 were incubated for 16 h at 24 °C. Analysis by SDS-PAGE with Coomassie staining. (**B**) Attempted dissociation of product from SnoopLigase2 using three different elution methods. HaloTag-SnoopLigase2 was captured with HaloLink-sepharose, followed by incubation with 3 M imidazole, glycine pH 2.5, or 100 μM SnoopTagJr:DogTag peptide. Analysis by SDS-PAGE with Coomassie staining. E1-3 are 50 μL elution fractions. B are the beads resuspended in 50 μL SDS elution buffer, to show the TG2x-DogTag2:SnoopTag2-sfGFP that had been retained on the beads.

.

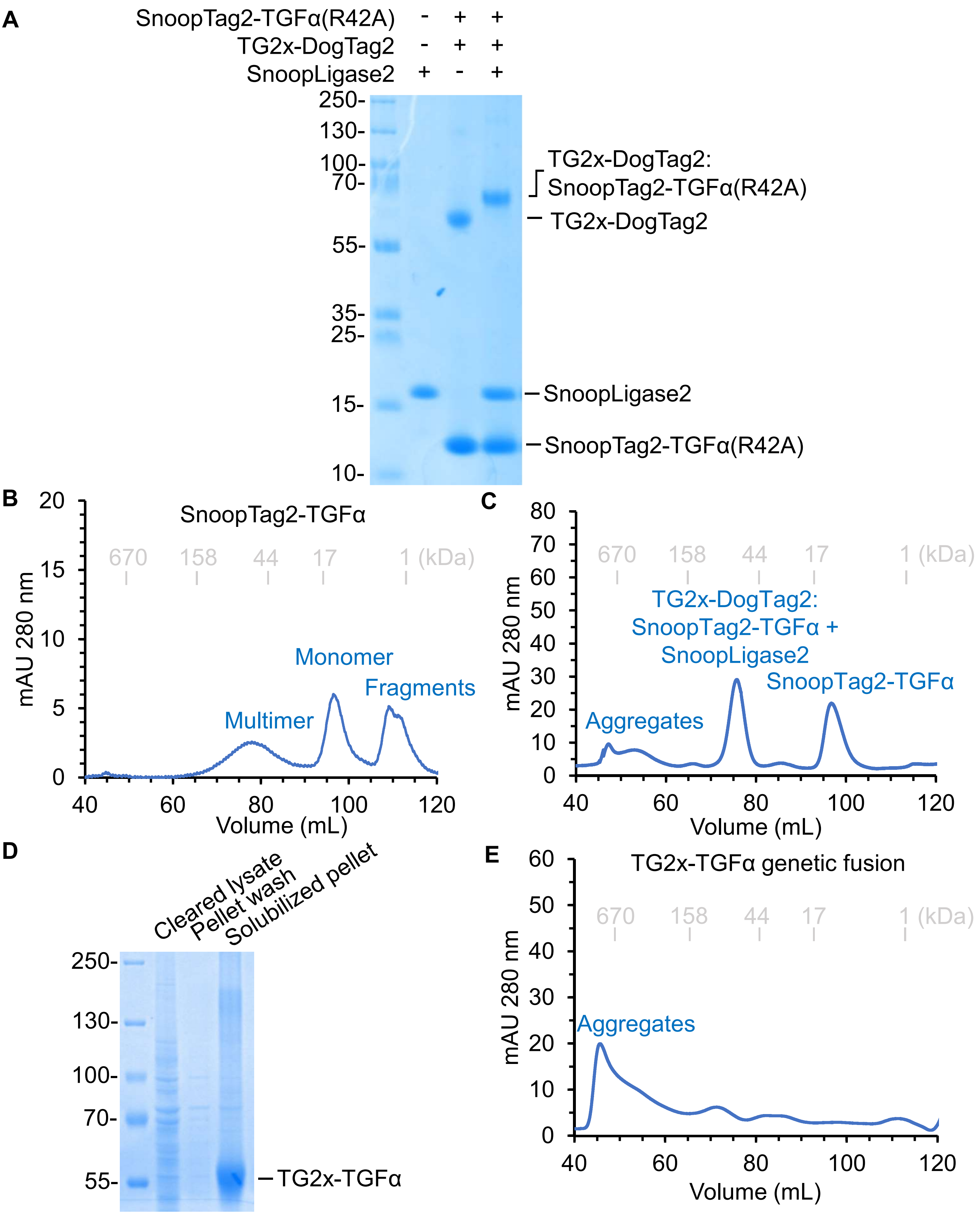

Figure S11. TG2x can be efficiently connected to TGFα by ligation but not genetic fusion. (**A**) Ligation of TGx and TGFα(R42A). 10 µM TG2x-DogTag2, 20 µM SnoopTag2-TGFα(R42A) and 15 µM SnoopLigase2 were incubated for 16 h at 4 °C before SDS-PAGE with Coomassie staining. (**B,C**) Size-exclusion chromatography. Solid blue line represents relevant sample and gray lines represent molecular weight markers. (**B**) SnoopTag2-TGFα after refolding from inclusion bodies. (**C**) SnoopLigase2 ligation of TG2x and TGFα units. 10 µM TG2x-DogTag2, 30 µM SnoopTag2-TGFα and 20 µM SnoopLigase2 were incubated for 16 h at 4 °C in PBS pH 7.4. (**D**) TG2-TGFα was expressed from inclusion bodies in *E. coli*, with samples taken for SDS-PAGE with Coomassie staining. (**E**) Size-exclusion chromatography of TG2-TGFα genetic fusion after denaturing and refolding shows high Mw aggregates.
